# Site-Specific Glycation of Human Heat Shock Protein (Hsp27) Enhances Its Chaperone Activity

**DOI:** 10.1021/acschembio.3c00214

**Published:** 2023-07-14

**Authors:** Somnath Mukherjee, Dominik P. Vogl, Christian F. W. Becker

**Affiliations:** † University of Vienna, Faculty of Chemistry, Institute of Biological Chemistry, Währinger Strasse 38, 1090 Vienna, Austria; ‡ Vienna Doctoral School in Chemistry, Währinger Strasse 42, 1090 Vienna, Austria

## Abstract

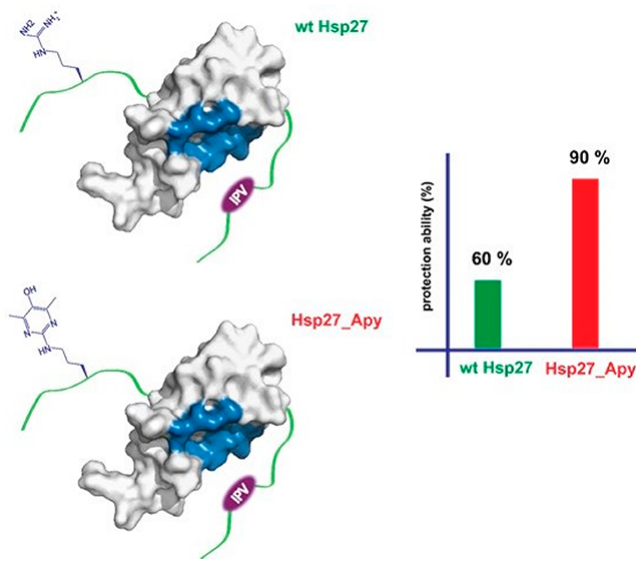

Non-enzymatic posttranslational modifications are believed to affect at least 30% of human proteins, commonly termed glycation. Many of these modifications are implicated in various pathological conditions, e.g., cataract, diabetes, neurodegenerative diseases, and cancer. Chemical protein synthesis enables access to full-length proteins carrying site-specific modifications. One such modification, argpyrimidine (Apy), has been detected in human small heat shock protein Hsp27 and closely related proteins in patient-derived tissues. Thus far, studies have looked into only artificial mixtures of Apy modifications, and only one has analyzed Apy188. We were interested in understanding the impact of such individual Apy modifications on five different arginine sites within the crucial N-terminal domain of Hsp27. By combining protein semisynthesis with biochemical assays on semisynthetic Hsp27 analogues with single-point Apy modification at those sites, we have shown how a seemingly minimal modification within this region results in dramatically altered functional attributes.

## Introduction

Posttranslational modifications (PTMs) of proteins constitute a constantly expanding field that mostly deals with regulatory processes in eukaryotic cell biology.^1−3^ At the core of these crucial processes lies a variety of rather complex pathways in which many proteins are subjected to precisely orchestrated and spatiotemporally regulated covalent modifications. Typically, such modifications, involving covalent attachment of functionalities, small molecules, and proteins (e.g., acetylation, glycosylation, phosphorylation, ubiquitination, etc.) at specific residues of a target protein, are tightly regulated via specific enzymes. They can lead to subtle changes in the structure–activity attributes of a large range of membrane and soluble proteins.^4,5^ Any perturbation of this delicate balance often induces aberrant protein modification, the consequences of which often lead to the onset of pathological conditions.

Apart from these heavily studied enzyme-mediated PTMs, another class of analogous protein modifications collectively known as non-enzymatic posttranslational modifications (nPTMs) exists, where typically an electrophilic, reactive metabolite reacts with nucleophilic side chains of amino acids (e.g., lysine, arginine, and histidine).^6^ Most likely, the best studied group of nPTMs consists of advanced glycation end (AGE) and advanced lipoxidation end (ALE) products.^7^ Although the generation and biological implication of such non-enzymatic modifications are far from being completely understood, they have been mostly considered as biomarkers in the context of cellular stress, molecular aging, and disease etiology. These modifications are in many cases irreversible and thus can heavily affect the structure and function of long-lived proteins.^8^ It is thus extremely important to comprehend the underlying molecular pathways and concentration thresholds leading to the impairment and even loss of activity of certain proteins as a result of nPTMs.

**Figure 1:**
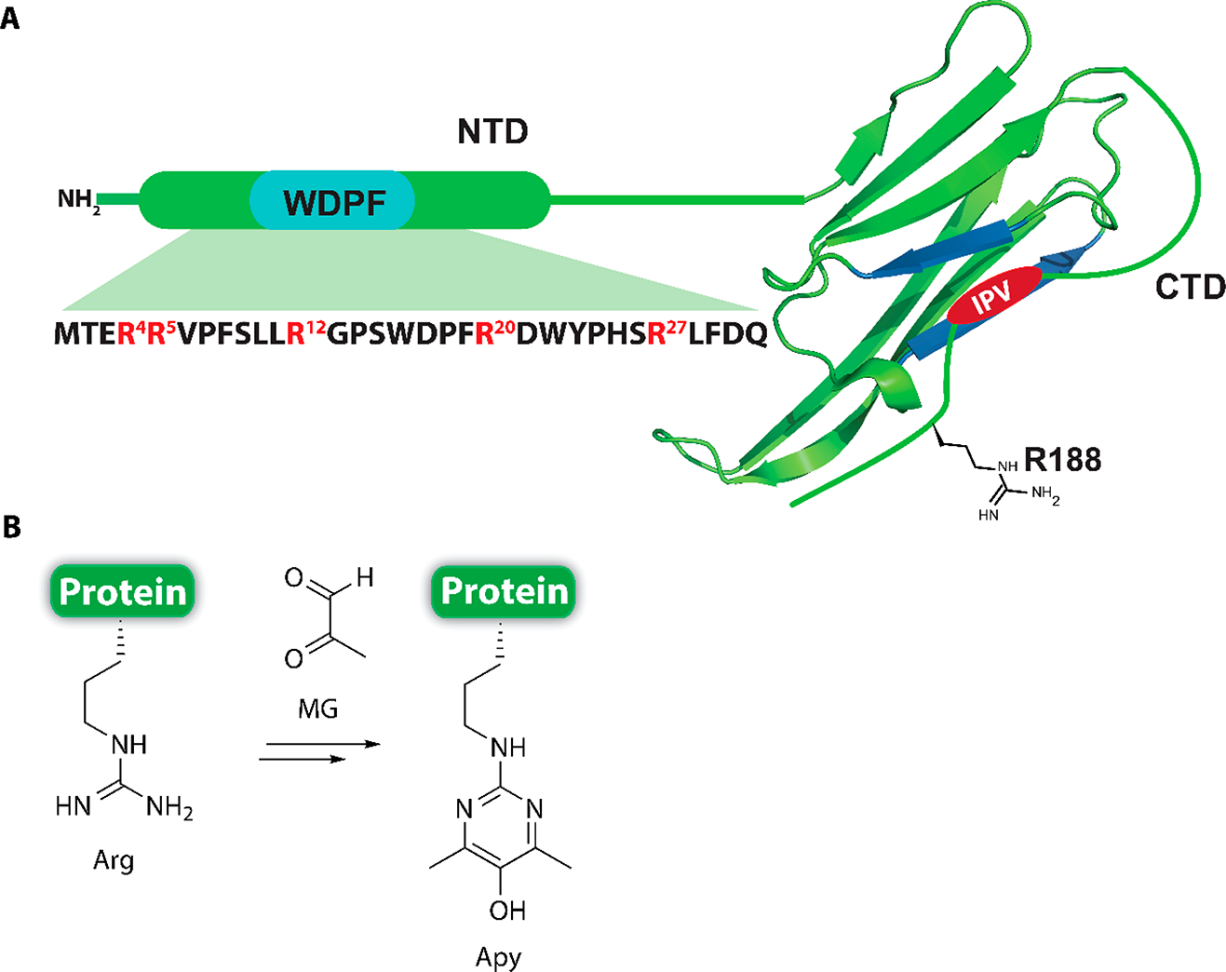
Salient features of Hsp27. (A) Domain architecture of Hsp27 highlighting the amino acid sequence within the distal NTD. Arginine residues chosen for the single-point Apy modification are colored red. The functionally important IPV motif (red) within the CTD interacts with the β4−β8 groove (deep blue) of the ACD. The illustration was created utilizing the reported crystal structure (Protein Data Bank entry 4mjh). (B) Generation of argpyrimidine (Apy) via the methylglyoxal (MG) modification of arginine.

Human small heat shock proteins (sHsps) make up a group of proteins typically in the molecular weight range of 12–43 kDa, which execute crucial functions in cellular proteostasis.^9−13^ Their primary role under cellular stress conditions is to retain a number of intracellular proteins prone to misfolding, in a “folding compatible” soluble state via an ATP-independent pathway until ATP-dependent chaperones such as Hsp70 and Hsp90 can refold them into their native states.^14^ One of the most studied members of this superfamily is human heat shock protein 27 (Hsp27), which is also the most abundant protein and ubiquitously expressed in all organs and tissues. Apart from serving as part of the first line of defense under cellular stress conditions, or “cellular paramedics”, they are also known to play important roles in apoptosis and protecting cells from oxidative stress. Interestingly, all sHsps in various species share a common structural feature, the well-structured α-crystallin domain (ACD), which represents an β-sandwich immunoglobulin-like fold flanked by a short and flexible C-terminal domain (CTD) and also a longer N-terminal domain (NTD) of variable length and composition (Figure 1A). Over the past three decades, evidence has accumulated indicating that Hsp27 is one of the major targets of methylglyoxal (MG)-derived AGEs.^15−17^ Interestingly, although as many as 16 arginine residues in Hsp27 are vulnerable to MG modification resulting in the formation of the fluorescent AGE argpyrimidine (Apy) (Figure 1B), arginine 188 within the CTD was so far found to be the residue most abundantly converted into Apy.^18^ In addition, we found evidence of a methylglyoxal modification of conserved arginine residue 12 in the NTD in three different sHsps: Hsp27, αA-crystallin, and αB-crystallin.^19^ These findings are based on proteomics data and analysis with the anti-Apy antibody mAb3C^19^ detecting the Apy modification(s) in Hsp27 on the only arginine within the short, flexible, and solvent-exposed CTD.^20^ Gathering structural evidence for such AGEs at the molecular level is highly important, but the dynamic and polydisperse oligomerization states of Hsp27 combined with its intrinsically disordered NTD and CTD make this challenging beyond the well-defined central α-crystallin domain. In the recent past, however, primarily on the basis of cryo-electron microscopy and/or cross-linking/mass spectrometry, nuclear magnetic resonance (NMR) spectroscopy, and molecular modeling, crucial insights have been provided.^21,22^ Apart from this inherent difficulty, deciphering a more site-specific impact of the Apy modification on the structure–activity attribute of Hsp27 is challenging as generating an Apy-modified sample via simple incubation with a suitable electrophilic reagent inevitably generates a heterogeneously modified mixture complicating all downstream analysis.^16,23^ Additionally, there are currently no genetic manipulation techniques for incorporating Apy as a noncanonical amino acid at a predetermined site(s).^24,25^

**Figure 2:**
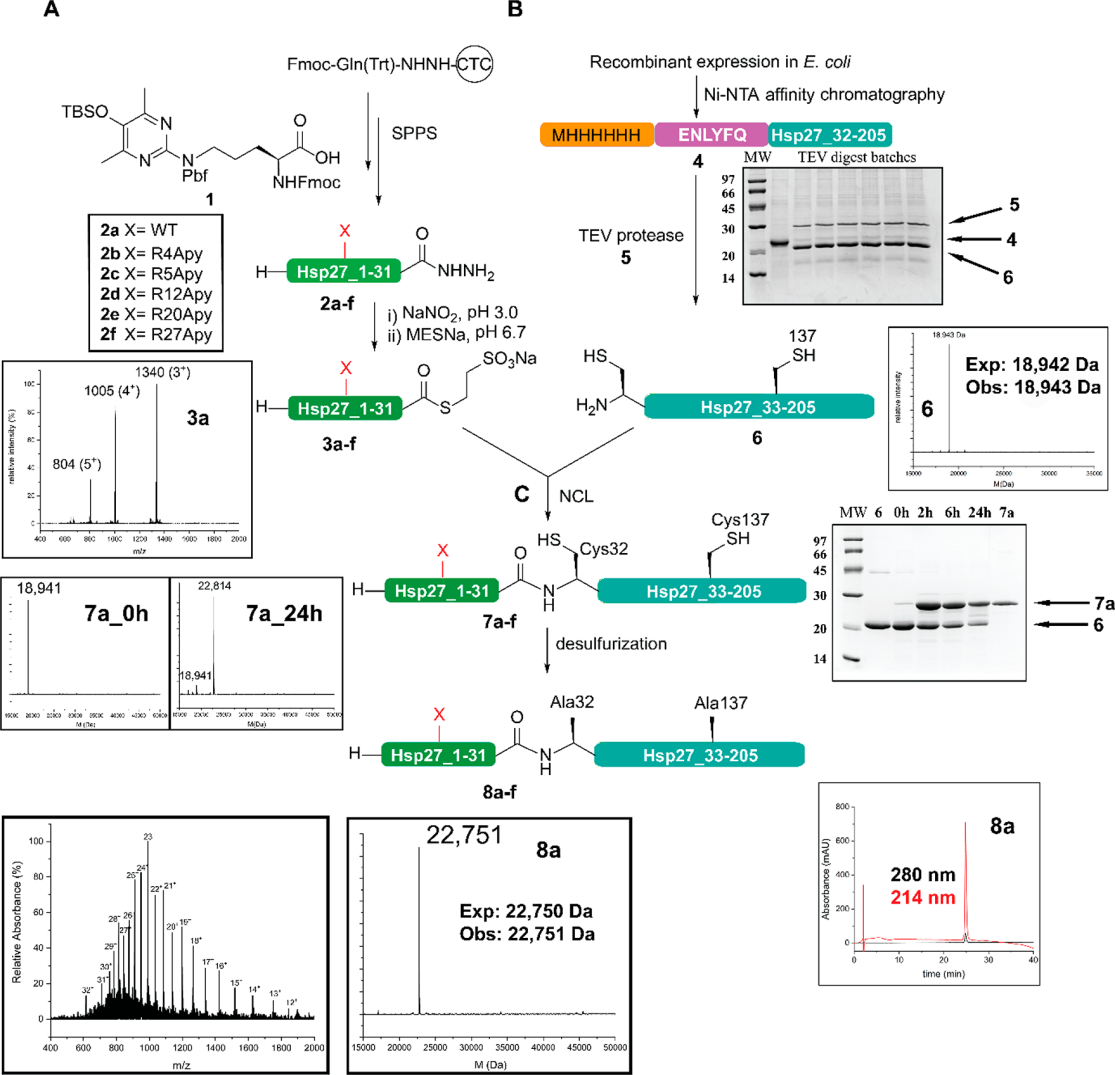
Semisynthesis of site-specifically Apy-modified full-length Hsp27. (A) Fmoc/*t*-Bu-based SPPS utilizing building block **1** generated the site-specifically Apy-containing thioesters **3a**–**f**. (B) Recombinant expression of a fusion construct **4** followed by enzymatic removal of the affinity tag provided the unmodified segment **6** required for the subsequent (C) native chemical ligation (6 M Gdn·HCl, 0.2 M NaP_i_, 300 mM MESNa, 50 mM TCEP, pH 7.8, and 37 °C) and desulfurization (6 M Gdn·HCl, 0.2 M NaP_i_, 250 mM TCEP, 100 mM MESNa, 20 mM VA-044, pH 7.2, and 37 °C) to furnish the final products **8a**–**f**, which were further characterized by ESI-MS and RP-HPLC.

In pursuit of the development of a robust semisynthetic pathway to access such site-specifically Apy-modified Hsp27, we previously developed and utilized a novel Apy building block **1** suitably functionalized for solid phase peptide synthesis (SPPS) (Figure 2A).^26,27^ This tailor-made and orthogonally protected unnatural amino acid building is also compatible with Fmoc/*t*-Bu SPPS and native chemical ligation (NCL).^28−30^ Our previous results showed that the single-point mutation R188Apy within the CTD leads to the impairment of the in vitro chaperone activity against citrate synthase along with a partial disruption of the native oligomeric structure of Hsp27.^27^ Other recent reports on Hsp27 have additionally established the fact that depending on the nature and site of modifications within the same CTD, very different effects on Hsp27 activity are observed. In one such report, the authors showed that an *O*-GlcNAc modification in the vicinity of the IXI/V motif in a number of sHsps prevented their ability to intramolecularly compete with substrate binding, leading to an enhancement of the anti-amyloid chaperone activity against both α-synuclein and Aβ(1–42).^31^

In contrast, the long (∼90 residues) and variable NTD of Hsp27 has remained an enigmatic factor in sHsp function.^32−34^ Despite its intrinsically disordered nature,^35^ a number of specialized techniques based on NMR spectroscopy and X-ray crystallography have provided some insights,^36−38^ consolidating the importance of the entire NTD as well as various shorter motifs within it for oligomerization, substrate selectivity, and chaperone activity.^19,39,40^ Although most of the earlier literature describes interactions involving the β4−β8 groove of the ACD and the conserved IXI/V motif of the CTD (Figure 1A),^37,41^ more recent reports have unravelled various intra- and intermolecular interactions involving unique stretches of the NTD with the ACD, both at the dimer level.^35^ Considering our previous findings that showed the clear impact of a single Apy modification on Hsp27 activity, we wanted to investigate the important NTD with its high prevalence of Arg sites and the known modification site Arg 12.^19^ Therefore, we replaced five arginine residues with argpyrimidine within the distal NTD (residues 1–31) by employing an NCL strategy.

## Results and Discussion

### Synthetic Strategy for Accessing Site-Specifically Apy-Modified Hsp27

To generate multiple site-specifically modified variants of Hsp27 with Apy in different positions within its N-terminal domain, we employed a native chemical ligation (NCL) strategy in which a synthetic N-terminal peptide was linked to a larger recombinantly produced part (Figure 2). Because the only available native cysteine (Cys137) in Hsp27 is not suitably located for such a strategy, we decided to utilize Gln31-Ala32 as the ligation site for which a simple point mutation of Ala32 to Cys would enable us to perform the ligation, followed by desulfurization to convert the non-native cysteine to alanine (Figure 2C).^42,43^ At the same time, native Cys137 would also be converted to an Ala residue. We and others have shown that this C137A mutation has no impact on the oligomeric structure and chaperone activity of Hsp27 in our experiments.^44^

To generate the N-terminal 31-mer peptide thioester comprising parts of the NTD, we assembled the corresponding peptide hydrazides **2a**–**f** on 2-chlorotrityl chloride (CTC) resin via Fmoc/*t*-Bu SPPS while utilizing building block **1** at predetermined sites. Following global deprotection and purification of the peptide hydrazides, they were converted to stable MESNa (sodium 2-mercaptoethanesulfonate) thioesters (**3a**–**f**) following a previously reported protocol (Figure 2).^45^ Although it is feasible to synthesize a more reactive MPAA (4-mercaptophenylacetic acid)-based thioester, one needs to be cautious while readjusting the pH at different steps as such reactive MPAA thioesters tend to hydrolyze faster around pH 7.0.^45^ Here, preparing the more stable MESNa thioester provides higher yields and improved control over subsequent ligations.

To generate recombinant protein segment **6**, fusion construct **4** containing a tobacco etch virus (TEV) protease recognition sequence was designed, which also contained an N-terminal His tag for affinity purification (Figure 2). Following recombinant expression in *Escherichia coli* and subsequent purification of this construct via Ni-NTA affinity chromatography, it was subjected to TEV-protease digestion to give Hsp27_32–205 (**6**) (Figure S8). This fragment was further subjected to purification via preparative reverse phase high-performance liquid chromatography (RP-HPLC) and characterized via analytical RP-HPLC and electrospray ionization mass spectrometry (ESI-MS) (Figure S9). Purified product **6** was obtained in high yield (15 mg/L of 2YT culture) and high analytical purity (>95%).

### Native Chemical Ligation of Synthetic Hsp27_1–31 MESNa Thioester with Recombinant Hsp27_32–205

The NCLs between the thioester peptides (**3a**–**f**) and **6** were performed under denaturing conditions using MESNa (sodium 2-mercaptoethanesulfonate) as the thiol additive followed by metal free desulfurization (MFD). These ligations were typically carried out at a peptide thioester (**3a**–**f**) concentration of 2 mM and recombinant segment **6** concentration of 1 mM, in a volume of 500–800 μL. The progress of the ligation was monitored via analytical RP-HPLC, ESI-MS, and SDS–PAGE (Figure S10). The ligations reached near completion over the course of 24 h, following which the crude ligation mixture was subjected to semipreparative RP-HPLC purification and lyophilization to afford a yield of 3–4 mg (50–70%) for all of the ligation products **7a**–**f**. It is worth mentioning here that these NCL reactions could also be performed using MPAA (4-mercaptophenylacetic acid) as the thiol additive to convert MESNa thioesters **3a**–**f** into more reactive MPAA thioesters, which shortens the reaction time to 3–4 h to reach completion. However, one must be cautious about eliminating any trace amount of MPAA as it interferes with the subsequent desulfurization step. In addition, MPAA thioesters also co-elute with some of the ligation products, complicating their purification. It should be noted that there are other ligation additives such as trifluoroethanethiol (TFET) that allow “one-pot” ligation–desulfurization chemistry.^46^ However, because we observed quantitative consumption of the starting material after overnight incubation under optimized conditions, we did not explore further additives. To be consistent, we performed all of our ligations with MESNa, as the use of MPAA did not further improve the final yield of the ligation product.

To convert the non-native Cys32 back to native Ala32, a desulfurization was carried out on the ligation products using VA-044 as the water-soluble radical initiator and MESNa as the hydrogen donor in the presence of neutral TCEP. Under these conditions, we achieved a clean conversion of the ligation product to the desired desulfurized product in 2–3 h. The reaction mixtures were then subjected to semipreparative RP-HPLC purification and lyophilization to afford a yield of ∼3 mg of each semisynthetic Hsp27 variant (∼40% overall yield over two steps for **8a–f**). The final semisynthetic products hence obtained were analyzed via analytical RP-HPLC and ESI-MS (Figures S11–S16).

The lyophilized final products were subjected to a refolding procedure in 40 mM HEPES·KOH (pH 7.5) at a concentration of ∼1 mg mL^–1^, followed by incubation at 25 °C for 3 h. The refolding efficiencies for all of the variants were quantitative. Refolded proteins then were characterized to understand the structural and functional consequences of the Apy modification.

### Protein Folding and Function

**Figure 3:**
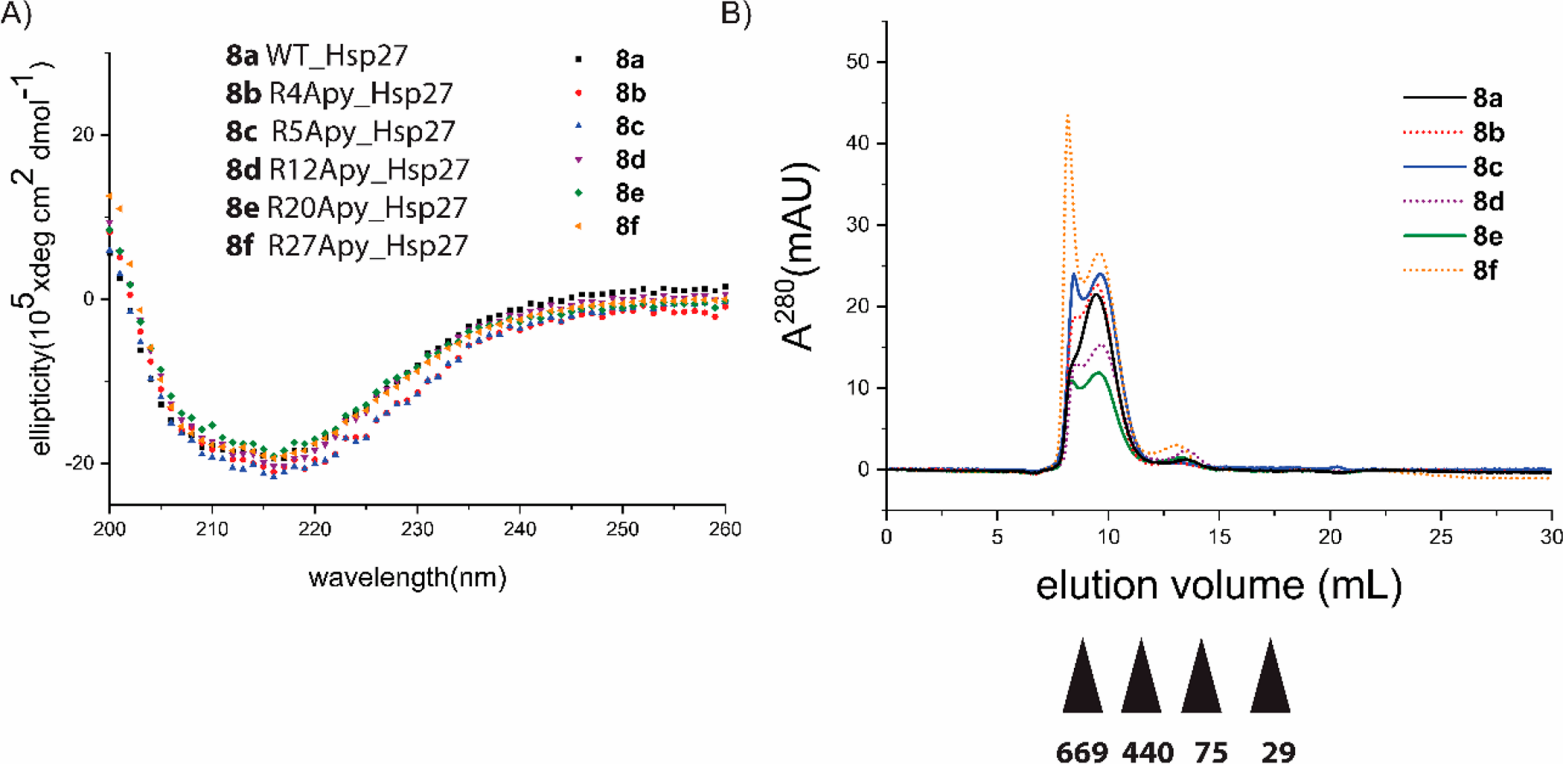
Biophysical characterization of semisynthetic Hsp27 analogues. (A) Overlay of the far-ultraviolet circular dichroism spectra of folded wild type **8a** and site-specifically Apy-modified Hsp27 **8b**–**f**. For the sake of clarity, the identity of the Hsp27 variants is also included. (B) Size-exclusion chromatography profile of folded Hsp27 samples **8a**–**f**. The elution volume and the molecular weight (in kilodaltons) of the standard proteins used for calibration are shown as triangles.

Circular dichroism spectra of the wild type as well the modified analogues of Hsp27 showed the characteristic broad minima around 215 nm indicating a well-defined immunoglobulin G-like β-sandwich structure as reported in the literature (Figure 3A and Figure S19).^47^ This means that the site-specific conversion of arginine to argpyrimidine within the unstructured NTD of Hsp27 does not perturb secondary structure.

To understand the impact of single-Apy modifications on the oligomeric assembly of Hsp27, we analyzed size-exclusion chromatography (SEC) profiles of **8a**–**f** on a Superdex-200 column. The folded wild type (WT) as well as the Apy-modified Hsp27 samples showed double maxima around 9–10 mL, indicating that Apy modifications within the distal NTD of Hsp27 do not impact the oligomeric assembly significantly on a level that can be detected by SEC (Figure 3B). This observation differs from our previous finding for a semisynthetic Hsp27 variant site-specifically Apy modified at position 188 [close to the beginning of the C-terminal region (CTR) and the IXI/V motif], in which partial dissociation of the larger oligomers into smaller assemblies as well as into the monomer was observed.^27^ This difference can be explained by the role of N- and C-terminal domains in oligomerization. Upon close inspection, we observe some subtle differences in the elution pattern of our semisynthetic variants. Although Hsp27 is known to exist in a dynamic equilibrium between several oligomeric states, WT variant **8a** showed the least distinct higher-order oligomeric assembly. Variants **8b**–**e** showed almost similar levels of higher-order oligomeric species, whereas **8f** seems to form even more higher-order oligomers. Taken together, single-Apy modifications cause a slight shift toward the formation of higher-order oligomeric assemblies, which we cannot directly relate to changes in chaperone activity (see below).

**Figure 4:**
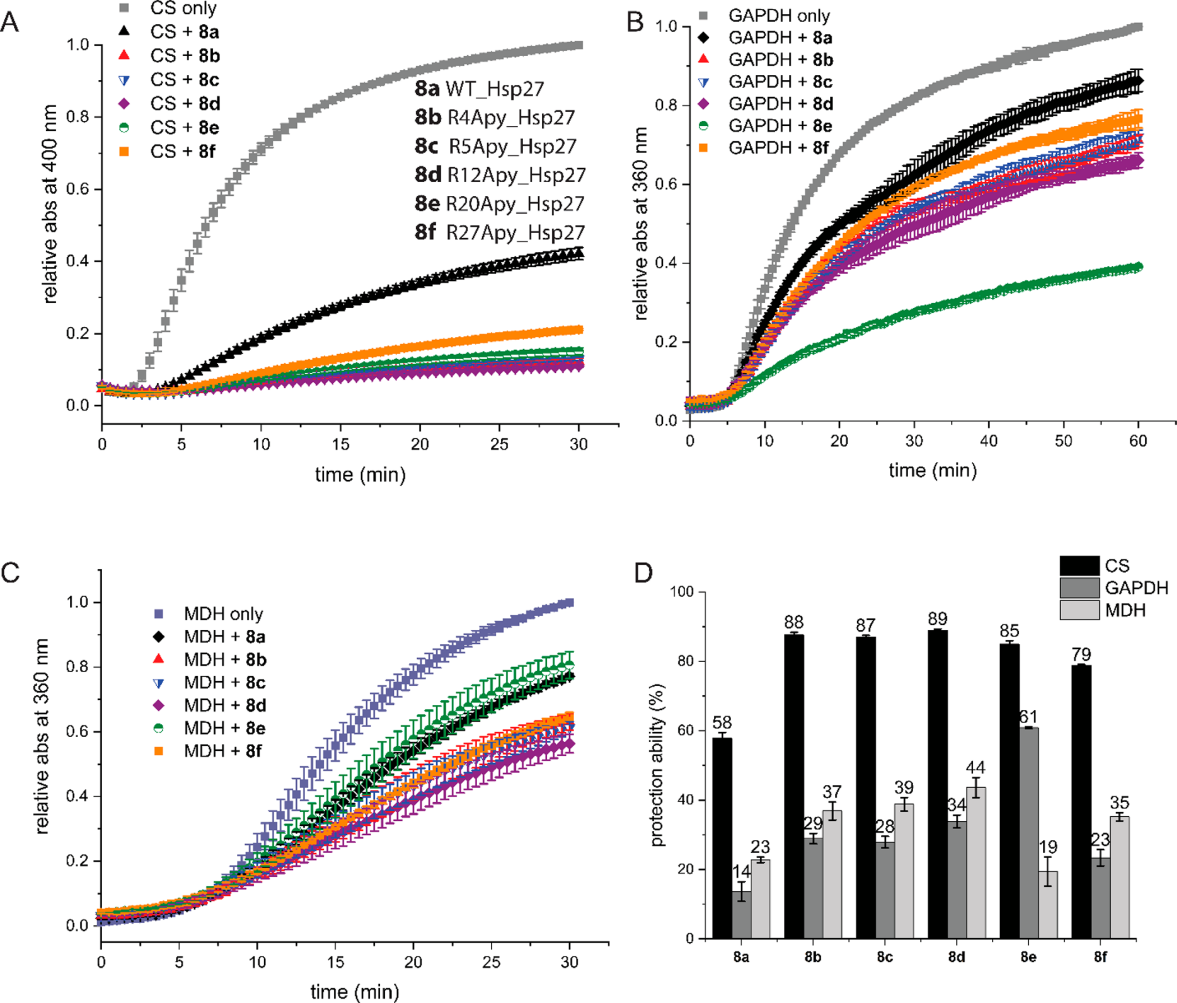
Biochemical characterization of semisynthetic Hsp27 analogues. (A) Citrate synthase (CS; 2 μM) was incubated at 45 °C in the absence or presence of 0.45 μM Hsp27 analogues **8a**–**f**. (B) Glyceraldehyde 3-phosphate dehydrogenase (GAPDH; 3 μM) was incubated at 45 °C in the absence or presence of 0.6 μM Hsp27 analogues **8a**–**f**. (C) Malate dehydrogenase (MDH; 2 μM) was incubated at 45 °C in the absence or presence of 0.25 μM Hsp27 analogues **8a**–**f**. (D) Chaperone activity expressed as percentage protection with respect to amorphous aggregation in the absence of a chaperone.

The consequences of site-specific Apy modifications on Hsp27 activity were assessed on the basis of chaperone activity on a variety of client proteins, starting with citrate synthase (CS). The latter was previously used as a model client protein by us and others.^27,48^ We subjected CS to temperature-induced aggregation at 45 °C in the absence and presence of the WT (**8a**) and Apy-modified Hsp27 variants (**8b**–**f**). Aggregation was followed by monitoring the scattering at 400 nm. WT **8a**, as expected, delayed the aggregation of CS and suppressed it by ∼60% (Figure 4A). Quite interestingly, all five Apy-modified analogues **8b**–**f** also delayed the aggregation and suppressed it to an even greater extent (∼80–90%) when compared to WT **8a**, indicating again that single-point Apy modifications have a drastic impact on the chaperone activity of Hsp27 (Figure 4A). It has been well documented that the chaperone activity of various sHsps on different client proteins is strongly dependent on the particular sHsp–client protein pair.^48^ To clarify whether this enhanced chaperone activity is specific to citrate synthase, we decided to test our Apy-modified Hsp27 variants **8b**–**f** with different client proteins. This was essentially to test our Apy-modified Hsp27 variants against more challenging client proteins. To this end, we selected two client proteins: glyceraldehyde 3-phosphate dehydrogenase (GAPDH) and malate dehydrogenase (MDH), for which wild type Hsp27 was shown to be active, but to a lower extent than for CS.^38,48^ To our delight, we observed that against GAPDH, variants **8b**–**d** and especially **8e** were found to be more active than WT **8a**, and against MDH, variants **8b**–**d** were more active than the WT **8a**, inferring that the enhanced chaperone activity was not merely a client protein-specific observation (Figure 4B–D).

**Figure 5:**
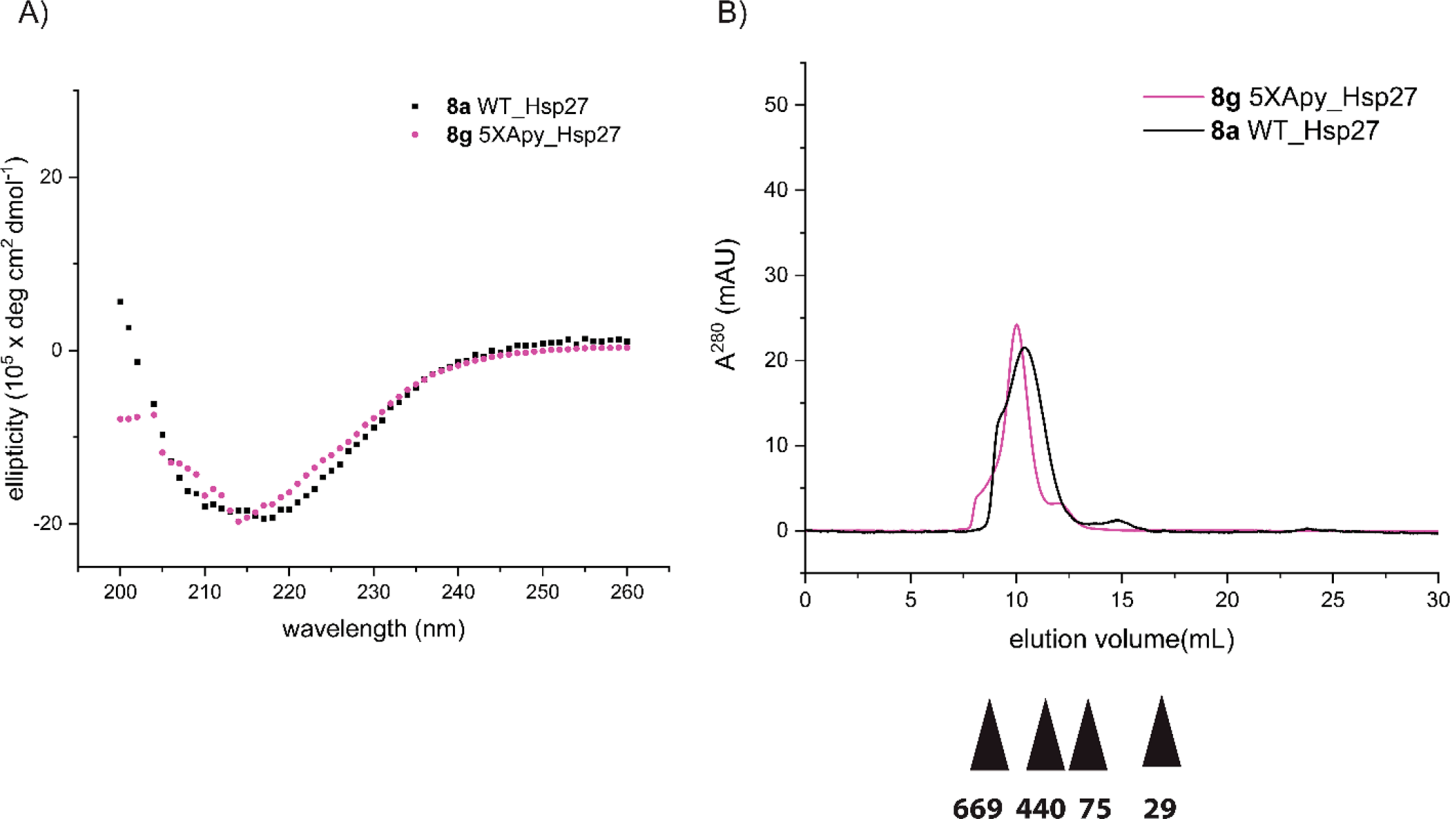
Biophysical characterization of semisynthetic Hsp27 analogues **8a** and **8g**. (A) Overlay of the far-ultraviolet circular dichroism spectra of folded wild type **8a** and penta-Apy-modified Hsp27 **8g**. (B) Size-exclusion chromatography profile of folded Hsp27 samples **8a** and **8g**. The elution volume and molecular weight (in kilodaltons) of the standard proteins used for calibration are shown as triangles.

**Figure 6:**
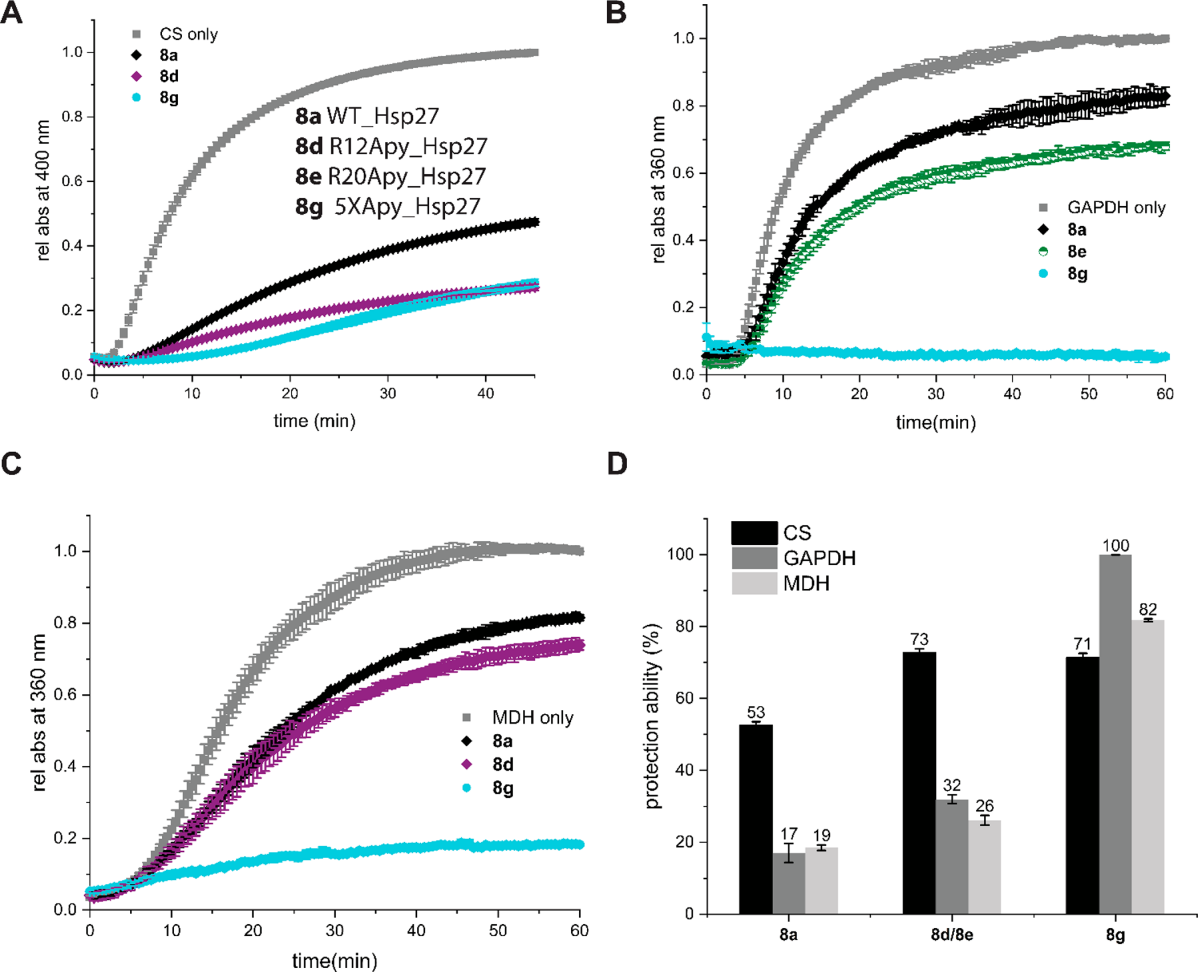
Comparison of in vitro chaperone activity with those of the client proteins. (A) Citrate synthase (CS; 2 μM) was incubated at 45 °C in the absence or presence of 0.45 μM Hsp27 analogues **8a**, **8d**, and **8g**. (B) Glyceraldehyde 3-phosphate dehydrogenase (GAPDH; 3 μM) was incubated at 45 °C in the absence or presence of 0.6 μM Hsp27 analogues **8a**, **8e**, and **8g**. (C) Malate dehydrogenase (MDH; 2 μM) was incubated at 45 °C in the absence or presence of 0.25 μM Hsp27 analogues **8a**, **8d**, and **8g**. (D) Chaperone activity expressed as percentage protection with respect to amorphous aggregation in the absence of a chaperone.

Intrigued by our finding, we wanted to understand whether there is an additive effect of Apy modifications. We synthesized Hsp27 variant **8g** with all five previously analyzed arginine sites converted into Apy (Figure S17). This variant gave a CD spectrum similar to those of the WT and singly modified Hsp27 variants, in addition to the fact that the minimum at 215 nm is more defined than for the unmodified or singly modified variants (Figure 5A). This indicates a slight change in secondary structure, which is further supported by the SEC profile of **8g** (Figure 5B). This profile is very similar to that of WT Hsp27 and shows no peak for very large oligomeric species as found for variants **8b**–**f**. This difference in the oligomerization state for the penta-modified variant could be one explanation for the dramatically increased chaperone activity toward several client proteins as described below. To directly compare chaperone efficiency against a particular client protein, we employed WT **8a** along with penta-modified Hsp27 **8g** and variant **8d/8e** that we earlier found to be the most effective against a particular client protein in question. Against CS, no further enhancement of chaperone activity due to the additional four Apy modifications was found (Figure 6A). However, we observed a significant increase in activity against GAPDH and MDH (68% and 56%, respectively), indicating a “superchaperone” effect against these client proteins (Figure 6B–D).

**Figure 7:**
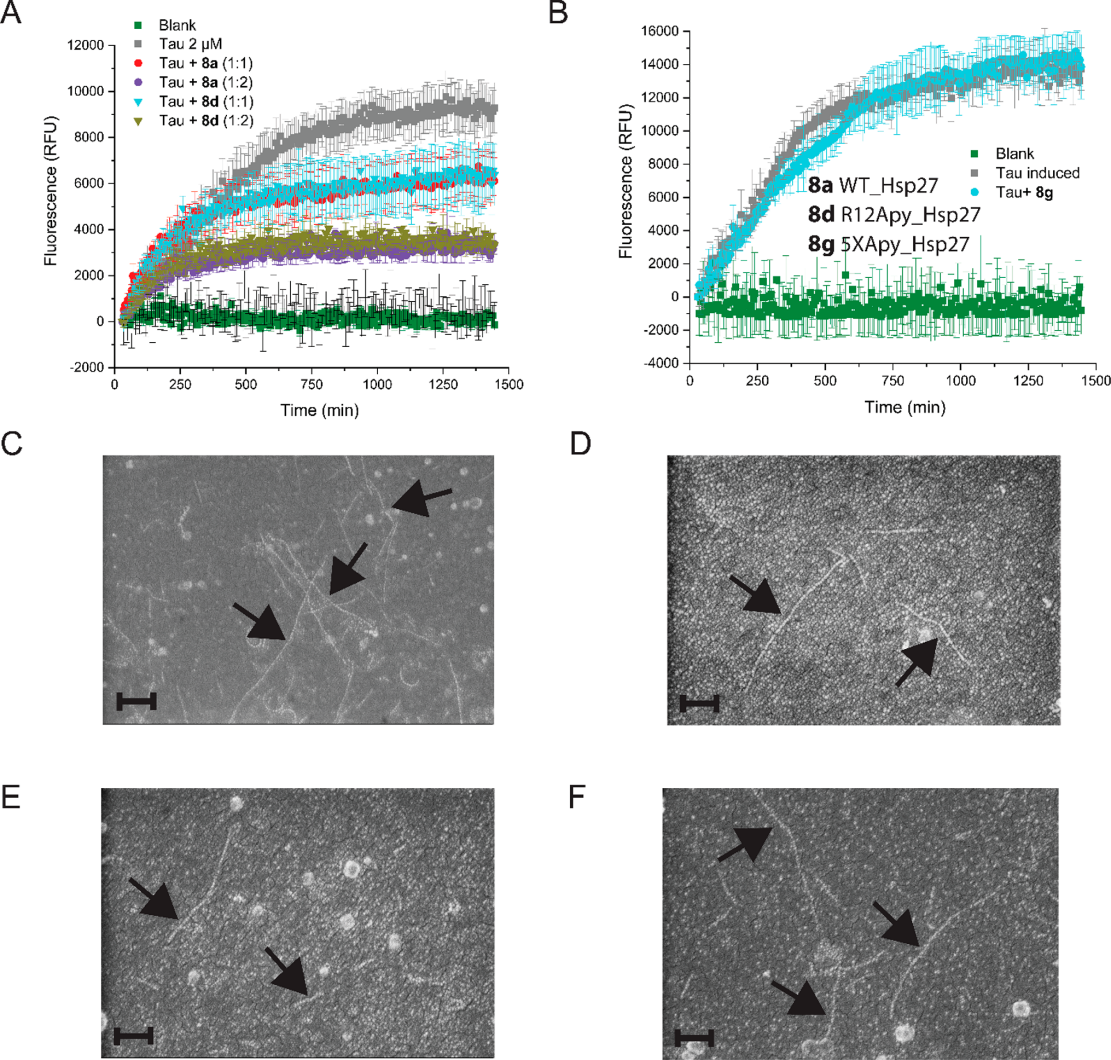
In vitro chaperone activity assay of **8a**, **8d**, and **8g** against tau as a model client protein known to undergo amyloidogenic aggregation. The aggregation was monitored via ThT fluorescence (excitation at 444 nm and emission at 490 nm) as well as scanning electron microscopy. (A) Activity comparison between **8a** and **8d** at two different doses (1:1 and 1:2 tau:**8a** or tau:**8d**). (B) Activity of penta-modified version **8g** (1:1 tau:**8g**). (C) Scanning electron microscopy (SEM) image from the assays with tau only (induced). (D) Image from the assays with tau and **8a**. (E) Image from the assays with tau and **8d**. (F) Image from the assays with tau and **8g**. Scale bars of 200 nm. Black arrows mark the tau fibrils.

**Figure 8:**
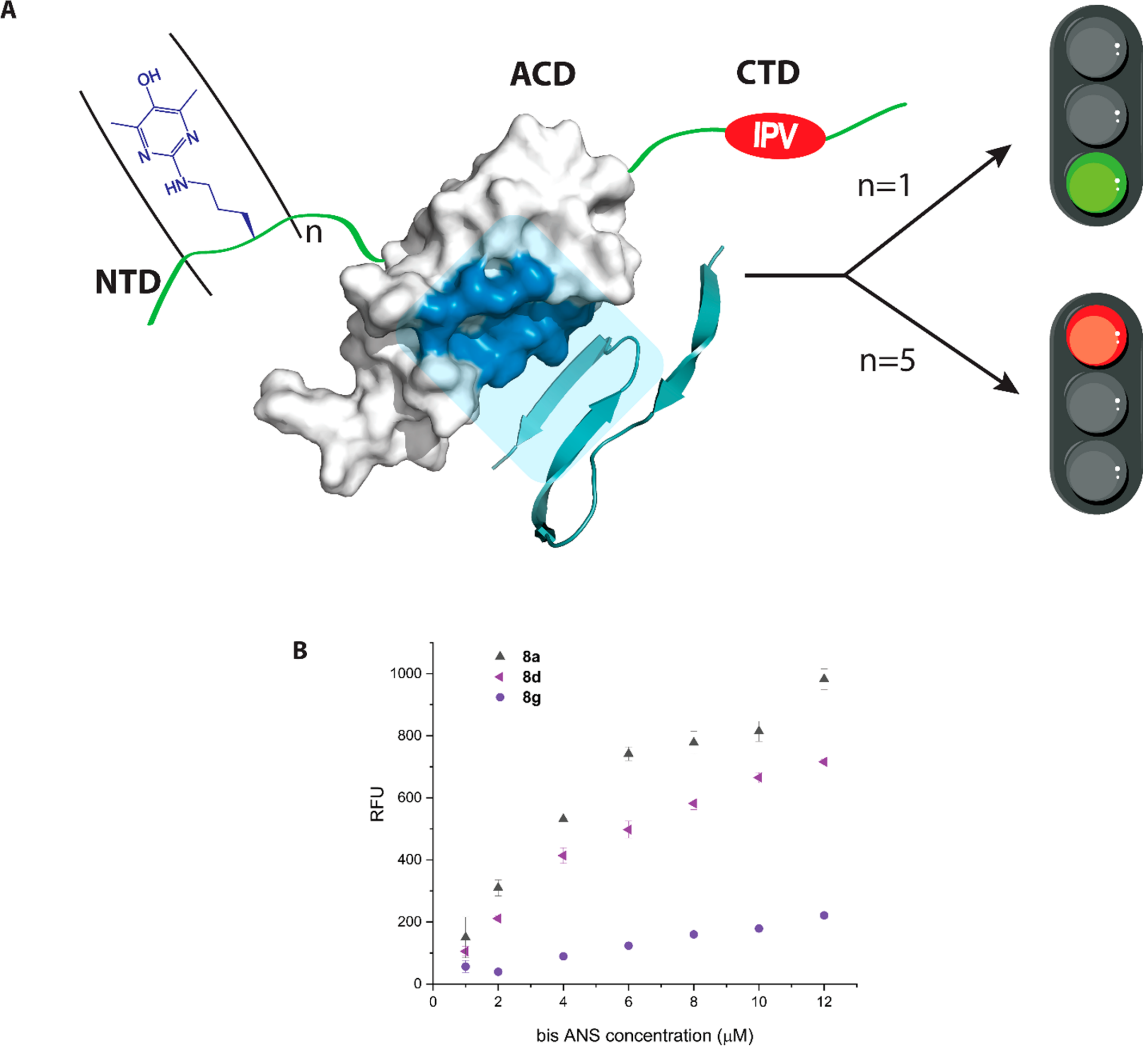
(A) Impact of Apy modification on the chaperone activity of Hsp27 against an amyloidogenic client protein. Single-site modification (*n* = 1) causes no apparent change in the activity as the hydrophobic binding patch (colored dark teal) is still available for binding to the client protein (colored teal), while multiple modifications (*n* = 5) presumably cause certain structural reorganizations of Hsp27 resulting in the abolishment of anti-amyloid chaperone activity. The illustration was created utilizing the reported crystal structures of Hsp27 (Protein Data Bank entry 4mjh) and Aβ(1–42) (Protein Data Bank entry 2mxu). (B) Titration curves for **8a**, **8d**, and **8g**. A chaperone concentration of 4.4 μM was mixed with increasing concentrations of bis-ANS (1–12 μM), and the fluorescence emission intensity was recorded at 495 nm using an excitation wavelength of 385 nm. The data are represented as an average of three measurements, including the standard error of the mean.

Inspired by our exciting chaperone assay results against client proteins that undergo amorphous aggregation under heat stress, we sought to test our site-specifically Apy-modified Hsp27 variants against an amyloid-forming client protein, as well. Here, we utilized tau4, a known client protein of Hsp27, for which a set of key interactions pertaining to the chaperone activity of Hsp27 has been described, mainly on the basis of NMR experiments.^49,31,50,51^ While subjecting recombinant full-length tau4 to in vitro aggregation, we monitored thioflavin T (ThT) fluorescence in the presence of WT **8a** and in the presence of Apy-modified Hsp27 **8d**. Both were similarly effective in preventing aggregation (Figure 6A). Penta-modified version **8g**, on the contrary, did not show any chaperone activity at all against tau4 (Figure 7B). We also analyzed the supernatants of ThT assays by scanning electron microscopy (SEM). In accordance with the ThT assay data, we observed for samples with tau only and with tau and **8g** (panels C and F, respectively, of Figure 7) a higher abundance of tau fibrils (black arrows) than in samples with tau and **8a** and with tau and **8d** (panels D and E, respectively, of Figure 7). This supports the notion that chaperone activity is highly client protein specific and is a consequence of specific protein–protein interaction modes as well as the aggregation pathway of the client protein. Thus, conclusions with respect to certain classes of client proteins can be drawn only when assessing enhancement or impairment of chaperone activities, if one can procure a very detailed picture of the specific chaperone–client protein interaction interface. Interestingly, an earlier report by Mainz et al. indicated that for another closely related sHsp αB-crystallin, fibril-forming client proteins such as Aβ(1–40) preferentially bind to a hydrophobic edge of the central ACD, while amorphously aggregating client proteins are captured via the partially disordered NTD of the chaperone.^52^ Another work published during the course of preparation of this paper suggested a mechanism in which monomerization of sHsp leads to an inhibition of amyloid fibril elongation.^53^ This plausibly explains why our Apy-modified chaperones exhibit differential chaperone activity between the two classes of client proteins that we tested. We envision that the single-point modifications within the NTD did not perturb the structure of the chaperone (Figure 8A) to compromise the hydrophobic binding patches on the ACD. However, with five modified sites in **8g**, a significant electrostatic and structural reorganization could cause the loss of crucial client protein binding patches on the ACD, resulting in the loss of chaperone activity.

### Surface Hydrophobicity Analysis of Hsp27 Variants

The intrinsically disordered N-terminal domain of Hsp27 together with the protein’s polydispersity and highly dynamic exchange between smaller and larger oligomers renders any detailed structural insight quite challenging. Due to this behavior, we wanted to discern in an indirect way whether an altered surface hydrophobicity originating from the single and/or multiple modifications on Hsp27 somehow correlates with changes in chaperone activity. We employed the environmentally sensitive probe bis-ANS, which while being nonfluorescent in its free state in solution, shows an enhancement in the fluorescence emission when bound to hydrophobic patches on the protein surface.^40,54^ We tested the overall most efficient singly modified chaperone **8d** together with WT **8a** and penta-modified version **8g**. As one can see clearly from the titration curves (Figure 8B), the affinity of **8d** for bis-ANS is marginally lower than that of **8a**. In contrast, penta-modified version **8g** binds significantly less bis-ANS than both **8a** and **8d**. It has been previously reported^40^ that Hsp27 possesses a solvent free accessible core with a high affinity for bis-ANS, most likely within the oligomers formed by this chaperone. In contrast to this, chaperones that exist predominantly in lower-order oligomeric structures, such as HSPB6 and HSPB8, lack such a core structure, which results in a lower affinity for bis-ANS. This finding is in accordance with the SEC data of our penta-modified variant **8g**, for which we observed a slight shift toward predominantly lower-order oligomers as compared to singly modified versions **8b–f**, which show a bimodal elution profile (Figures 3B and 5B). Taken together, the SEC and the bis-ANS binding data indicate that multiple Apy modifications cause a shift in the dynamic equilibrium toward the formation of lower-order oligomers, which results in significantly enhanced chaperone activity against a range of client proteins that undergo temperature-induced amorphous aggregation.

## Methods

### Native Chemical Ligation and Subsequent Desulfurization

For NCL, 5 mL of the degassed ligation buffer [6 M Gdn·HCl in 200 mM sodium phosphate buffer (pH 7.8)] was degassed via nitrogen/argon purging. Then, 500 μL of this degassed buffer was supplemented with 50 mM TCEP and 300 mM MESNa. Five milligrams (0.26 μmol) of Hsp27(32–205) **6** was then dissolved in 250 μL of this ligation buffer. The pH was then readjusted to 7.8 using NaOH solutions, and finally, 2 mg (0.52 μmol) of the respective thioester was added before the mixture was shaken at 37 °C under an inert atmosphere for 24 h. The ligation progress was monitored via LC-MS and SDS–PAGE. The desired ligation products (**7a**–**g**) were purified on a C4 semipreparative RP-HPLC column. The fractions containing the desired ligation product were combined to lyophilize them to a fluffy while solid. All of the lyophilized ligation products were either subsequently desulfurized or stored at −20 °C.

To perform the desulfurization, the lyophilized ligation product (∼3 mg) was dissolved in 200 μL of freshly degassed desulfurization buffer [6 M Gdn·HCl in 200 mM NaP_i_ (pH 7.2)]. To it was added 200 μL of a 500 mM neutral TCEP solution in the same buffer (final concentration of TCEP of 250 mM) followed by MESNa (6.5 mg, 100 mM, as a solid). The pH of this reaction mixture was readjusted to 7.2. Finally, 20 μL of a 400 mM solution of VA-044 in degassed water was added to the reaction mixture (final concentration of VA-044 of 20 mM), and the reaction tube was carefully flushed with nitrogen/argon gas and sealed. This reaction mixture was thereafter incubated at 37 °C for 2–3 h. Upon completion of the desulfurization, the crude mixture was subjected to semipreparative C4 RP-HPLC purification. Fractions containing the desired desulfurized ligation product were pooled, lyophilized to a white fluffy solid, and finally stored at −20 °C for further use.

### In Vitro Chaperone Activity Assay with Citrate Synthase

The assay was performed according to the literature method with minor modifications.^3^ Citrate synthase (CS, MW = 48,969 Da) from porcine heart was purchased from Sigma-Aldrich (Taufkirchen, Germany) as an ammonium sulfate suspension, centrifuged to remove most of the ammonium sulfate salts, and dialyzed against the storage buffer [50 mM Tris·HCl and 2 mM EDTA (pH 8)], to a final concentration of 20–30 μM. The accurate concentration of the CS stock solution was then determined using the bicinchoninic acid (BCA) assay, and the stock solution was subsequently flash frozen in liquid nitrogen in small aliquots (200–500 μL) and stored at −80 °C.

Amorphous aggregation of CS was monitored via measuring the absorbance at 400 nm in a SAFAS UVmc2 double-beam ultraviolet–visible spectrophotometer equipped with a temperature-controlled multicell holder (SAFAS, Monaco) in 700 μL quartz cuvettes (Hellma Analytics), in a 600 μL final volume in triplicate. The CS stock solution (obtained as described above) was diluted with 40 mM HEPES·KOH (pH 7.5) to a final concentration of 2 μM, and the resulting solution was used as such (control) or treated with Hsp27 variants (final concentration of 0.45 μM) followed by incubation at 45 °C while measuring the absorbance at 400 nm over 45 min (600 μL final volume in triplicate). Prior to the addition, all Hsp27 variants (lyophilized powders) were freshly dissolved in 40 mM HEPES·KOH (pH 7.5) buffer, the accurate concentrations of these primary stocks and that of CS were determined via the BCA assay, and another stock of all Hsp27 samples with a concentration of 1 mg mL^–1^ was prepared to refold them for 3 h at 25 °C. A baseline correction employing only the assay buffer [40 mM HEPES·KOH (pH 7.5)] was also performed. The raw data were exported from SAFAS software as a Microsoft Excel worksheet and processed using Microsoft Excel and OriginPro. The results were expressed as the average relative ultraviolet absorbance at 400 nm, where relative absorption at 400 nm = (absorption at 400 nm)/(maximal absorption at 400 nm by aggregating CS in the absence of a chaperone). The chaperone assays were repeated twice to obtain the final data.

### Estimation of Surface Hydrophobicity via Bis-ANS Binding

The binding of the nonfluorescent dye bis-ANS (4,4′-dianilino-1,1′-binaphthyl-5,5′-disulfonic acid, dipotassium salt) to Hsp27 samples was quantified on a 96-well plate format with a microplate reader (BioTek Synergy Mx). The accurate concentration of the chaperones was determined using the Pierce BCA Protein Assay Kit and adjusted to 1 mg mL^–1^ before the samples were subjected to refolding at 25 °C for 3 h in 40 mM HEPES·KOH (pH 7.5). The total volume (bis-ANS stock, buffer, and the refolded chaperone solution) in each well was 100 μL. The concentration of all of the Hsp27 samples was kept constant at 4.4 μM (final concentration). The concentration of bis-ANS was varied in the range of 1–12 μM. The bis-ANS fluorescence intensity was measured with an excitation wavelength of 385 nm (9 nm bandwidth) and an emission wavelength of 495 nm (17 nm bandwidth). An appropriate baseline correction was also performed without the chaperones. All data were acquired in triplicate, and the average of them was plotted along with the standard error of the mean. The binding assay was repeated twice to obtain the final data.

## Conclusions

Utilizing the potential of chemical protein semisynthesis, we have provided a pathway to access six site-specifically Apy-modified variants of Hsp27. Studying such glycated variants of small heat shock proteins and the impact of any site-specific glycation requires access to a homogeneously modified sample. We have demonstrated here that single-point Apy modifications of the small heat shock protein Hsp27 within the intrinsically disordered N-terminal domain cause an enhancement of chaperone activity against client proteins that undergo amorphous aggregation upon heat shock. The resulting “superchaperone” completely prevents the aggregation of certain client proteins in vitro. However, such modifications do not have any impact on chaperone activity against tau, a client protein that undergoes amyloid formation. This observation points out that the chaperone activity of small heat shock proteins is strongly dependent on the specific client protein–chaperone pair as a consequence of the specific modes of protein–protein interactions involved. Penta-modified Hsp27 variant **8g**, while behaving as an excellent chaperone against several amorphously aggregating client proteins, exhibits no chaperone activity toward amyloidogenic tau4.

Here, we have shown for Hsp27 and its Apy modifications that synthetic protein chemistry offers the opportunity to generate a group of site-specifically modified protein variants, including one variant with five modification sites in multimilligram amounts. Such multiply modified proteins are otherwise largely inaccessible, e.g., via the currently available genetic code expansion technologies. Detailed analysis of the secondary structure and oligomerization has helped us to identify different chaperone pathways and the impact of N-terminal Apy modifications on these pathways. However, gaining insights at the molecular level to identify crucial protein–protein interactions that can lead to such dramatic changes as a result of single-point Apy modification remains challenging. Nevertheless, this is an exciting goal especially in the context of diseases such as neurodegeneration, diabetes, and retinopathy or in aging. One could consider approaches such as segmental isotope labeling of Hsp27 to facilitate downstream analysis via two-dimensional nuclear magnetic resonance (2D NMR) spectroscopy to gain more spatial information about the chaperone–client protein interaction, even if larger oligomers are formed.^55^

## Supporting Information Available

Additional experimental details for all of the experiments mentioned in the text along with all of the materials and methods utilized, analytical characterization (analytical RP-HPLC and ESI-MS) of all of the intermediate compounds and building blocks for the native chemical ligation (such as the recombinant protein fragment, peptide thioesters, and final desulfurized semisynthetic Hsp27 variants), and monitoring of the progress of the native chemical ligation (via analytical RP-HPLC, ESI-MS, and SDS–PAGE) (PDF)
