# Supplementary material for: Site-Specific Glycation of Human Heat Shock Protein (Hsp27) Enhances Its Chaperone Activity
Source: ACS Chem Biol. 2023 Jul 14;18(8):1760–71. doi: 10.1021/acschembio.3c00214 (PMC10442856; doi:10.1021/acschembio.3c00214)
Supplement: Supplementary file 1 — cb3c00214_si_001.pdf [file cb3c00214_si_001.pdf]

Supporting Information

Site-specific glycation of human Heat Shock Protein (Hsp27) enhances its chaperone activity

Somnath Mukherjee[1], Dominik P. Vogl[1, 2], Christian F. W. Becker*[1]

[1]University of Vienna, Faculty of Chemistry, Institute of Biological Chemistry, Währinger Str. 38, 1090 Vienna, Austria; [2]Vienna Doctoral School in Chemistry, Währinger Str. 42, 1090 Vienna, Austria.

*Corresponding author: christian.becker@univie.ac.at

Materials and Methods

Chemicals. All the Fmoc (9-fluorenylmethyloxycarbonyl) and orthogonally protected amino acids used to assemble all the peptides via SPPS were bought from Iris Biotech GmbH, Marktredwitz, Germany. O-(6-chlorobenzotriazol-1-yl)-N,N,N',N'-tetramethyluronium hexafluorophosphate (HCTU), 1-[bis(dimethylamino)methylene]-1H-1,2,3-triazolo[4,5-b]pyridinium 3-oxid hexafluorophosphate (HATU), 6-chloro hydroxybenzotriazole (Cl-HOBt) were also purchased from GL Biochem, Shanghai, China. Diisopropylethyl amine (DIPEA), 1M hydrazine in anhydrous tetrahydrofuran, diisopropyl carbodiimide (DIC), ethyl (hydroxyimino) cyanoacetate (Oxyma), triisopropylsilane (TIPS), trifluoroacetic acid (TFA), anhyd tetrahydrofuran (THF), sodium nitrite, glacial acetic acid, sodium 2-mercaptoethane sulfonate (MESNa), guanidine hydrochloride (Gdn.HCl), Thioflavin T (ThT) and piperidine were purchased from Sigma-Aldrich. 2-Chlorotrityl chloride polystyrene (2-CTC) resin was purchased from Chemimpex, Illinois, USA. Solvents used such as *N,N*-dimethylformamide (DMF), dichloromethane (DCM), acetonitrile (ACN) were purchased from Biosolve (Valkenswaard, The Netherlands). All other chemical reagents and solvents were purchased in the highest available quality from the following companies if not noted otherwise: Sigma-Aldrich (Taufkirchen, Germany), Fisher-Scientific (Schwerte, Germany), TCI-Europe (Zwijndrecht, Belgium), VWR (Darmstadt, Germany), Roth (Karlsruhe, Germany), Merck (Darmstadt, Germany), Invitrogen (Darmstadt, Germany), J.T.Baker (Griesheim, Germany), NeoLab (Heidelberg, Germany) or Omnilab (Bremen, Germany).

Synthesis of the argpyrimidine building block 1

The building block **1**, utilized to prepare site-specifically modified peptide hydrazides **2a-g** was synthesized in 5 g scale following the previously reported procedure.[1]

General method for the synthesis of the peptide hydrazides on solid support

Manual solid phase peptide synthesis (SPPS) was done utilizing 2-chlorotrityl chloride resin loaded with a hydrazine linker, on 0.05 mmol scale employing polypropylene syringes fitted with a frit (10 mL). All the Fmoc and orthogonally protected amino acids (5.0 equiv) were coupled after pre-activation (for 3 min) with 0.1M HCTU (O-(1H-6-Chlorobenzotriazole-1-yl)-1,1,3,3-tetramethyluronium hexafluorophosphate) (4.76 equiv) in the presence of diisopropylethylamine (10.0 equiv) and 6-chloro 1-hydroxybenzotriazole (Cl-HOBt) (5.0 equiv) for 30 min. The amino acids following prolines were coupled for a longer 45 min period.

To functionalize 2-CTC resin with hydrazine, 500 mg of the resin (typically 0.6 mmol) was subjected to swelling in 5 mL of THF (anhyd) for 1 h, the solvent was then discarded and to the syringe similar volume of 1M hydrazine in THF was taken to stir it for 1h, the resin was thereafter thoroughly washed with DCM then DMF. To cap the unreacted active sites, the resin was treated with 1:1 mix of DMF-MeOH containing a few drops of DIPEA for 15-20 min. Following washes with DMF, the C-terminal amino acid (Fmoc-Gln(Trt)-OH in our case) was coupled to the hydrazine loaded resin (Fmoc-Xaa-OH, HATU, HOAt, DIPEA: 5.0:4.76:5.0:10.0), 30 min coupling time, performed twice. Following all these procedures, the resin was thoroughly washed with DMF, DCM, DCM-MeOH (1:1), MeOH to dry it overnight in a vacuum desiccator.

After assembling the entire peptide, the Fmoc deprotected dry resin (~ 100 mg) was subjected to global deprotection using the cocktail TFA (trifluoroacetic acid)/triisopropylsilane/water-92.5/5.0/2.5 for 2.5 h. The crude peptide hydrazide was precipitated using ice-cold diethyl ether and washed twice with it before dissolving it in acetonitrile/water (1:1) containing 0.1% TFA, to lyophilize. The outcome of the SPPS was determined via LCMS and RP-HPLC. Finally, it was purified via preparative RP-HPLC. The fractions containing pure peptide hydrazide were pooled up, lyophilized and stored at -20 °C for the following step.

General method for the conversion of the peptide hydrazide to peptide thioester

30 mg (7.7 μmol) of the peptide hydrazide was dissolved into 3.7 mL of 200 mM NaPi buffer containing 6 M guanidine hydrochloride (Gdn.HCl), pH 3 and cooled to -15 °C. 1.2 mL of a freshly prepared 1 mg/mL $NaNO_2$ solution in water was added dropwise to it and the reaction mixture was stirred at -15 °C for 15 min. Then, 3.3 mL of a 9.5 mg/mL sodium 2-mercaptoethanesulfonate (MESNa) solution in 200 mM NaPi- 6M guanidine hydrochloride (Gdn.HCl) buffer, pH 3.0 was added to it, the pH was raised carefully to 6.7 with 1M NaOH and the mixture was stirred at room temperature for 20-30 min. The product was purified with a preparative C4 RP-HPLC column and the fractions containing the desired peptide thioester were pooled and lyophilised. All the purified and lyophilized peptide thioesters were stored at -20 °C until the following step.

RP-HPLC and mass spectrometry

Analytical LC-MS was performed on a Waters Auto Purification HPLC/MS system (equipped with 3100 Mass Detector, 2545 Binary Gradient Module, 2767 Sample Manager and 2489 UV/Visible Detector). Mass spectra were acquired by electrospray ionization (ESI) operating in positive ion mode. Separation was achieved with a Kromasil C4 column (300-5-C4, 150 × 4.6 mm, 5 µm particle size) or a Kromasil C18 column (300-5-C18, 150 × 4.6 mm, 5 µm particle size) at a flow rate of 1 mL/min while running a linear gradient of 5 to 65% of buffer B (ACN + 0.05 % TFA) in buffer A (ddH2O + 0.05 % TFA) over 10 min. Analytical RP-HPLC was conducted on a Dionex Ultimate 3000 instrument using a Kromasil C4 column (300-5-C4, 150 x 4.6 mm, 5 µm particle size) or a Kromasil C18 column (300-5-C18, 150 × 4.6 mm, 5 µm particle size) at a flow rate of 1 mL/min running a linear gradient of 5 to 65% of buffer B (ACN + 0.08% TFA) in buffer A (ddH2O + 0.1% TFA) over 30 min, monitoring absorption at 214 and 280 nm. Semi-preparative RP-HPLC was performed on a Waters Purification HPLC system (Waters 2545 Quarternary Gradient Module, Waters 2489 UV/Visible Detector & Waters Fraction Collector III) using a Kromasil C4 column (300-10-C4, 250 x 10 mm, 10 µm particle size) at a flow rate of 5 mL/min running a linear gradient of 25 to 70% buffer B (ACN + 0.08 % TFA) in buffer A (ddH2O + 0.1 % TFA) over 40 min, monitoring absorption at 214 and 280 nm. Preparative RP-HPLC was performed on a Waters Purification HPLC system (see above) using a Kromasil C4 column (300-10-C4, 250 x 21.2 mm, 10 µm particle size) at a flow rate of 10 mL77676/min running a linear gradient of 5 to 45% or 25 to 70% buffer B (ACN + 0.08 % TFA) in buffer A (ddH2O + 0.1 % TFA) over 40 min, monitoring absorption at 214 and 280 nm.

Materials and general procedures for cloning and expression of Hsp27_32-205 fusion construct

The GeneJET plasmid miniprep DNA Purification kit System for isolation of plasmids from *E. coli* cells and T4 DNA ligase were purchased from Thermo Fisher Scientific (Schwerte, Germany). Restriction enzymes (NdeI and XhoI) were purchased from Promega (Mannheim, Germany). Alkaline phosphatase (Shrimp), agarose were purchased from Sigma-Aldrich, and 100 bp and 1kb DNA ladders from Invitrogen. Chemically competent *E. coli* strains XL1-Blue, BL21(DE3) and Rosetta 2(DE3) were from Strategene and Invitrogen, respectively. Oligonucleotide synthesis and gene sequencing were performed by Eurofins Genomics AT GmbH (Vienna, Austria). All buffers were prepared with Milli-Q water (ddH2O). Protein analysis was carried out by SDS-PAGE using 15% acrylamide gels under reducing conditions. The protein molecular weight marker (ladder) kit was purchased from GE Healthcare (Freiburg, Germany). Gels were stained with Coomassie Brilliant Blue R-250. Centrifugal filtrations units were from Millipore (Amicon Ultra-15) or from Sartorius (Vivaspin 500). PD-10 Desalting Columns were from GE Healthcare (Freiburg, Germany). Protein concentrations were determined by the Pierce™ BCA Protein Assay Kit (Thermo Fisher Scientific), or by using NanoDrop 2000c (Thermo Fisher Scientific). For Hsp27 variants, the calculated extinction coefficient (40,450 $M^{-1}cm^{-1}$; ExpasyProtParam) was used in combination with the following MW values: Hsp27 (**8a**) 22,750 Da; Hsp27Apy (**8b-f**) 22,830 Da; 5XHsp27 (**8g**) 23,150 Da.

Subcloning, protein expression and purification

The oligonucleotide corresponding to fusion construct His tag_TEV recognition sequence-Hsp27_32-205 was subjected to purification via agarose gel electrophoresis, after a restriction digest of a custom made plasmid using NdeI and XhoI restriction enzymes was performed. This fragment was then sub-cloned into pET21a vector using the same restriction enzymes. The ensuing pET21a_His tag-TEV-Hsp27 (32-205) plasmid was then transformed into chemically competent *E. coli* Rosetta 2(DE3) host cells, which could then be stored as glycerol stocks (40% v/v in ddH_2O) at -80 °C or used to inoculate 600 mL of 2YT medium (16 g tryptone, 10 g yeast extract, 5 g NaCl/L of medium) supplemented with ampicillin (100 µg/mL) and chloramphenicol (30 µg/mL). This pre-culture was then shaken at 37 °C for 16 h before being used to inoculate a secondary culture (6 L) of the same medium. Protein expression was induced with 1 mM isopropyl β-D-1-thiogalactopyranoside (IPTG) during the log-phase ($OD^{600\ nm}$ = 0.6-0.8) and the resulting cell cultures were then shaken at 37 °C for 6 h. Subsequently, cells were harvested by centrifugation at 4 °C and the pellet was then washed with TBS buffer (50 mM Tris·HCl, 150 mM NaCl, pH 8.0) before being resuspended in the same buffer and lysed in a microfluidizer (Constant Systems TS), twice. The cell lysate hence prepared was then centrifuged at 4 °C to isolate the inclusion bodies containing the desired fusion construct, and the ensuing pellet was washed once with buffer (TBS with 0.1% Triton X-100) then centrifuged. After three cycles of washing with TBS and centrifugation, the pellet was solubilized in 50 mL of buffer A (6 M Gdn·HCl in 50 mM Tris·HCl, pH 8.0) overnight under vigorous stirring. For the final purification step via Ni-NTA affinity chromatography, the solubilized pellet was loaded onto a regenerated Ni-NTA column pre-equilibrated with buffer A. Afterwards, the column was washed thoroughly with buffer A to wash away all the undesired proteins lacking His-tag. To elute the protein of interest, an increasing gradient of buffer B (500 mM imidazole in buffer A): 0-100% in 60 min was employed. Fractions containing proteins were further analyzed via SDS-PAGE. All the fractions containing the desired protein were pooled and subjected to dialysis at 4 °C to remove imidazole and guanidine hydrochloride. The concentration of the protein was also measured via Nano-drop.

In order to generate the C-terminal EPL counterpart **6** having an N-terminal cysteine, the affinity tag had to be cleaved-off from the construct. To remove the affinity tag from the fusion construct, to the purified construct (conc ~ 1 mg/mL), urea was added to reach 2M concentration. This helps to partially denature the construct, as we realized from our previous experience that the N-terminal His-tag is not solvent exposed and thus a TEV cleavage without the addition of urea leads to only partial cleavage of the tag. To initiate the proteolysis, tris(hydroxypropyl) phosphine (THPP) (final concentration in the mixture 1 mM) followed by a solution of TEV protease (construct:TEV- 5:1) were added. This mixture was incubated at 4 °C for 6h.

In order to isolate the desired Hsp27_32-205 fragment **6** for the subsequent ligations, a batch Ni-NTA affinity purification was employed. The crude mixture was loaded onto the Ni-NTA column (5 mL) pre-equilibrated with buffer A, followed by binding for 1h. The flow through was collected. The column was then washed with TBS (4 mL x 3) to collect the washings. The collected flow

through was then subjected to semi-preparative C4 RP-HPLC purification. The fractions containing the desired product were pooled and lyophilized to store at -20 ℃ for further use.

Recombinant Expression of the flTau4 (full-length and longest variant of tau, 441 aa) was performed based on previously described protocols.[2] Therefore, a pET29b vector comprising a kanamycin resistance in an *E. coli* DE3 gold strain was used. Briefly, 2YT medium (16 g tryptone, 10 g yeast extract, 5 g NaCl/L of medium) were inoculated with an overnight culture until a starting OD_{600} = 0.2 was obtained. The cells were grown at 37 °C and 160 rpm for 90 min until an OD_{600} = 0.6 to 0.8. The over-expression of the desired flTau4 was induced by adding 1 mM IPTG (final concentration) and the expression was continued for 4 h (37 °C, 160 rpm). The progress of the over-expression was monitored by taking samples for SDS-PAGE at 0 h, 2 h and 4 h. After 4 h the cells were harvested by centrifugation for 20 min at 4000 g and 4 °C. After centrifugation the supernatant media was discarded and the pellets were processed further.
For cell lysis, the pellets (obtained from 6L of 2YT culture) were resuspended in 150 mL MES-buffer (50 mM $MES{\cdot}H_2O$, 5 mM DTT, pH 6.9) and lysed at 1.9 kbar at 10 °C (3x). Afterwards, the centrifuge tubes and the cell disruptor were washed with an appropriate amount of MES-buffer. The obtained cell suspension was centrifuged for 30 min at 48000 g. The supernatant containing flTau4 was transferred to a 500 mL round flask and stirred at 95 °C for 20 min until white precipitation was visible. Afterwards, the mixture was centrifuged for 45 min at 76500 g. The supernatant was filtered through a 0.22 µM Steritop-GP filter (Millipore) to remove possible arrears and samples for LC-MS (Waters Auto Purification LC-MS system in positive ion mode; 5% to 65% B in 10 min; Buffer A was Milli-Q H_2O + 0.05 % TFA and buffer B was HPLC-grade ACN + 0.05 % TFA) and SDS-PAGE were taken.
The filtered supernatant was purified directly using a preparative HPLC system. In order to purify flTau4 a gradient from 5% to 20% B in 5 min and 20% B to 50% B in 60 min was used. Product-containing fractions were pooled, frozen in liquid nitrogen and lyophilized. Due to the fact, that the purity of the obtained product was not sufficient for aggregation assays, the protein was purified again using a gradient from 5% B to 65 % B in 30 min (desalting: 10 min; flow rate: 10 mL/min) on the preparative HPLC system from Waters equipped with a Kromasil 300-10-C4 10 x 250 mm column (10 µM particle size). The fractions were analyzed as previously described, pooled and lyophilized (**Fig. S18**).

Circular dichroism (CD) spectroscopy

CD spectra were acquired using a Chirascan® Plus CD-spectrophotometer (Applied Photophysics, United Kingdom). The folding of all the Hsp27 samples were achieved via dissolving the lyophilized samples into 40 mM HEPES.KOH, pH 7.5 to a concentration of ~ 1 mg/mL followed by incubation at 25 ℃ for 3 h. Folded Hsp27 variants were buffer exchanged into 10 mM sodium phosphate buffer, pH 7.2 by Zeba™ Spin Desalting Columns (7K MWCO, 0.5 ml, Thermo Fisher Scientific). A final protein concentration of 0.1 mg/mL (NanoDrop, see above) in a micro cuvette (1 mm path length, Hellma Analytics, Germany) was used for each measurement. CD spectra were acquired at 25 °C from 200 to 260 nm in 1 nm steps. For each spectrum, 10 measurements were averaged and the background (buffer only) was subtracted. The raw data were exported from Pro-Data software as excel files and further processed using OriginPro.

Size exclusion chromatography (SEC)

All the Hsp27 samples **8a-f** were subjected to SEC on a ÄKTA Purifier system using a Superdex 200 (10 x 300 mm) column (both from GE Healthcare) equilibrated with 40 mM HEPES·KOH (pH 7.5) at 4 °C. For the estimation of the molecular weight, a combination of several proteins of the LMW and HMW calibration kits (GE Healthcare) were used: thyroglobulin, 669 kDa; ferritin, 440 kDa; conalbumin, 75 kDa; carbonic anhydrase, 29 kDa. The raw data were exported from UNICORN software as CSV and processed using OriginPro.

In-vitro chaperone activity assay

With malate dehydrogenase (MDH): The assay was performed in a similar way as above. MDH was purchased from Sigma-Aldrich (Taufkirchen, Germany) as lyophilized solid. The enzyme was dissolved into 2 mL of the assay buffer (phosphate buffered saline, pH 7.4), sterile filtered and divided into 200 µL aliquots to flash freeze them into liquid nitrogen and store at -80 °C. The final concentration of MDH was kept at 2 µM and that of Hsp27 samples at 0.25 µM. The amorphous aggregation was initiated in phosphate buffered saline (PBS), pH 7.4. The aggregation was observed at 360 nm over 30 min at 45 °C.

With glyceraldehyde 3-phosphate dehydrogenase (GAPDH): The assay was performed in a similar way as above. GAPDH was purchased from Sigma-Aldrich (Taufkirchen, Germany) as lyophilized solid. The enzyme was dissolved into 2 mL of the assay buffer (phosphate buffered saline, pH 7.4), sterile filtered and divided into 200 µL aliquots to flash freeze them into liquid nitrogen and store at -80 °C. The final concentration of GAPDH was kept at 3 µM and that of Hsp27 samples at 0.6 µM. The amorphous aggregation was initiated in phosphate buffered saline (PBS), pH 7.4. The aggregation was observed at 360 nm over 60 min at 45 °C.

Fibril formation assays with flTau4 in presence/absence of chaperones

The setup for the Thioflavin T (ThT) aggregation assays was based on previously described protocols by us and others.[2,4] For the aggregation assays the following stock solutions were prepared: 5x aggregation buffer: 50 mM HEPES, 500 mM NaCl, pH 7.4, 5 mM DTT (added freshly), 40 mM HEPES·KOH (pH 7.5), 2 mM ODS (octadodecyl sulfate) in 50 % (v/v) aqueous isopropanol and 100 µM Thioflavin T (ThT) in H_2O. All buffers were prepared, filtered (pore size: ≤ 0.22 µm) and either kept at 4 °C for short time storage or kept at – 20 °C for long time storage.
For the aggregation assays lyophilized aliquots of the proteins were freshly dissolved in appropriate volumes of H_2O (for flTau4) or 40 mM HEPES·KOH, pH 7.4 (for **8a**, **8d** and **8g**) on the day of the experiment. The protein concentrations were determined using a Pierce™ BCA Protein Assay Kit and afterwards adjusted to ~1.5 mg/mL (for flTau4) and 1 mg/mL (for **8a**, **8d** and **8g**). Refolding of the chaperones was performed by incubation on a table top shaker for 3 h at 25 °C and 350 rpm. Afterwards, 2 µM of flTau4 was pre-incubated in the presence/absence of

semisynthetic chaperones (2 µM/4 µM) for 30 min at 37 °C (350 rpm). Preincubation of Tau and the respective chaperones was performed in a final buffer composed of 80 µL 5x aggregation buffer, 18.2 µL/36.4 µL of HEPES·KOH and filled up with H_2O to a final volume of 390 µL. Afterwards, the aggregation was induced by adding 10 µL of ODS (or 10 µL of aqueous isopropanol for non-induced controls) and 44 µL of the ThT stock solution was added. The ThT assay was performed in 96-well microplates from Greiner Bio-One (black, non-binding, chimney-well). Each well contained a glass bead for improved mixing. The microplate was closed with adhesive foil to avoid loss of sample volume due to evaporation during the experiment. The aggregation process was monitored via ThT fluorescence (excitation = 444 nm; emission = 490 nm) at 37 °C with continuous shaking on a microplate-reader (BioTek Synergy Mx). Fluorescence emission values were recorded every 5 min for 24 h. Fluorescence emission values at 30 min were subtracted from the mean of the individual technical triplicate and set as a starting point for the aggregation kinetics.

Samples for scanning electron microscopy (SEM) were taken immediately after the end of the aggregation assay. For SEM 10 µL of the ThT assay solution was directly applied to a carbon supported copper grid, incubated for 3 min, 1x whisk-washed with 10 µL of H_2O and air-dried. The grids were fixed on the sample holder, sputter coated with gold nanoparticles under high vacuum (Bal-Tec SCD 005) and analyzed via SEM (Zeiss SEM Supra 55 VP).

a) b)

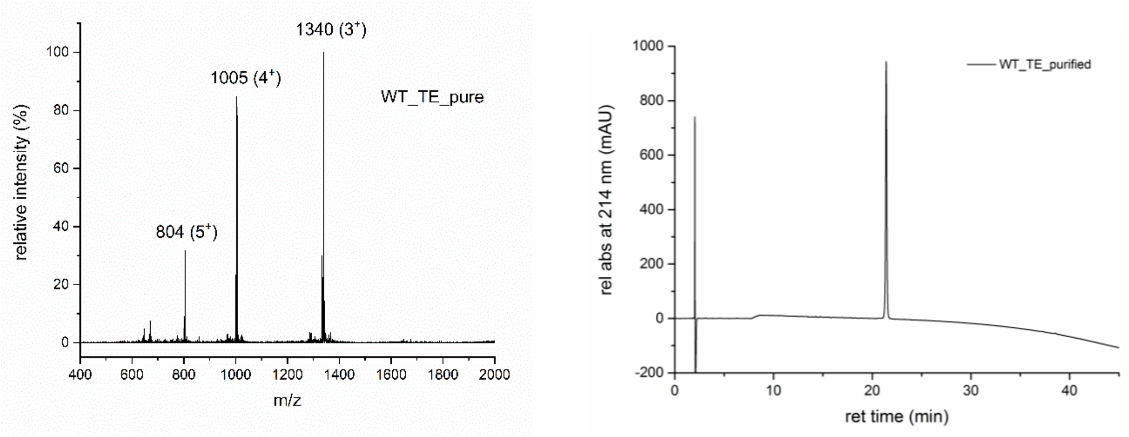

Figure S1. Characterization of the 31-mer wild-type thioester 3a. a) ESI-MS (Exp: 4016 Da, Obs: 4016 Da) b) analytical RP-HPLC profile

a) b)

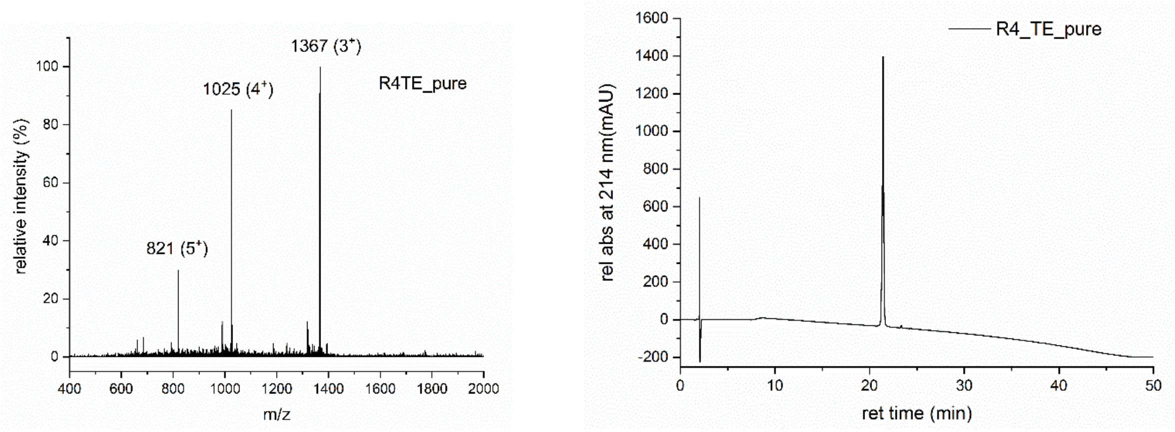

Figure S2. Characterization of the 31-mer R4Apy thioester 3b. a) ESI-MS (Exp: 4096 Da, Obs: 4096 Da) b) analytical RP-HPLC profile

a) b)

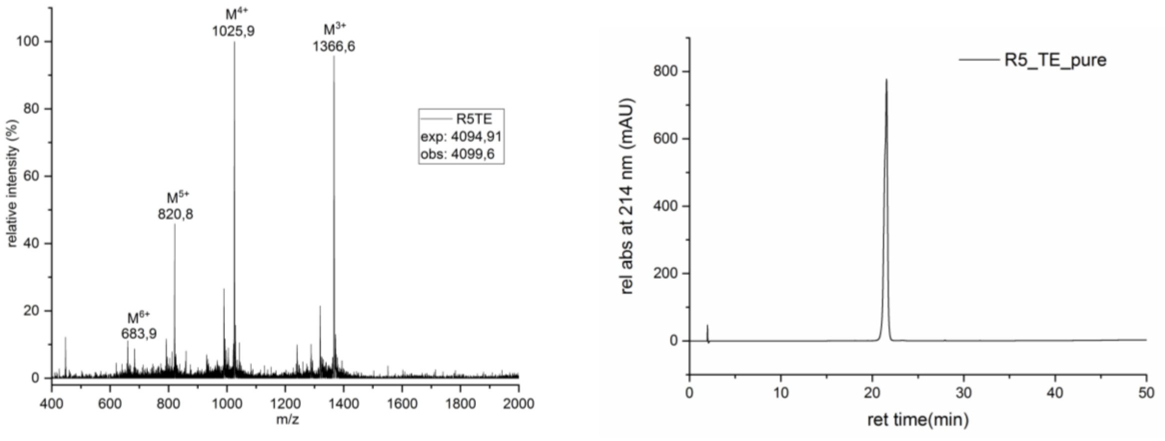

Figure S3. Characterization of the 31-mer R5Apy thioester 3c. a) ESI-MS (Exp: 4096 Da, Obs: 4100 Da) b) analytical RP-HPLC profile

a) b)

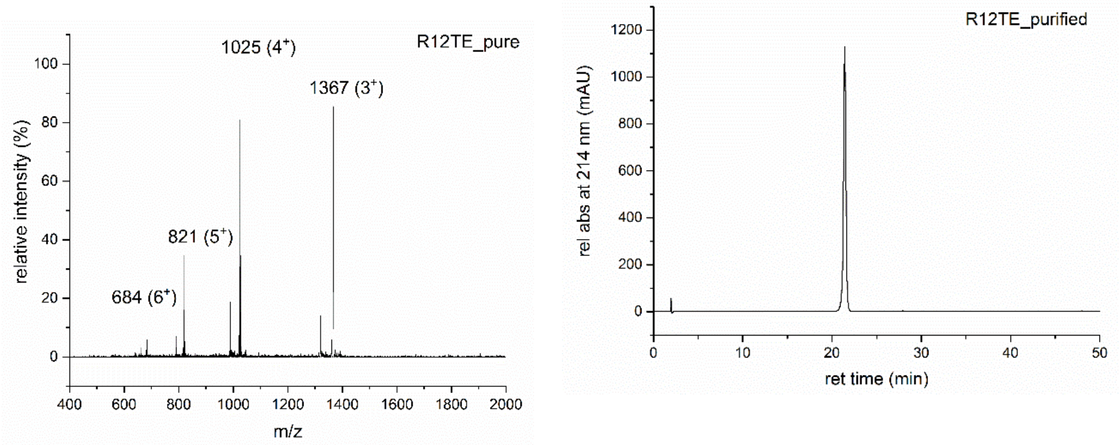

Figure S4. Characterization of the 31-mer R12Apy thioester 3d. a) ESI-MS (Exp: 4096 Da, Obs: 4098 Da) b) analytical RP-HPLC profile

a) **b)**

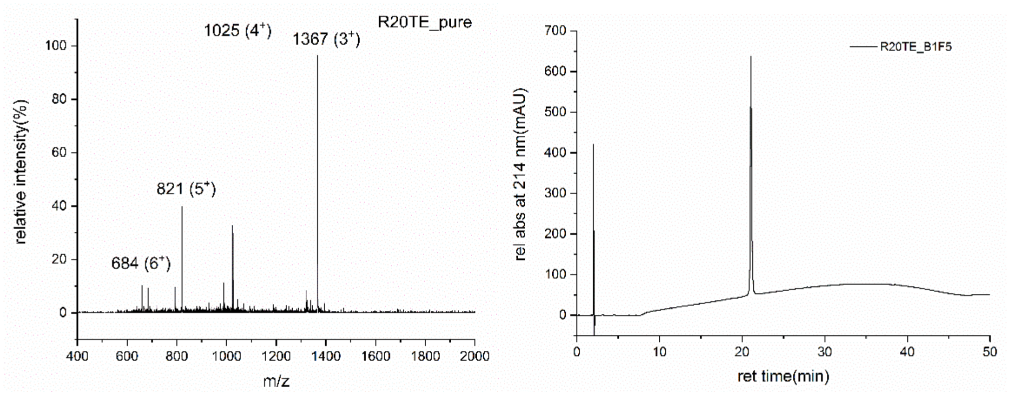

Figure S5. Characterization of the 31-mer R20Apy thioester 3e. a) ESI-MS (Exp: 4096 Da, Obs: 4098 Da) b) analytical RP-HPLC profile

a) **b)**

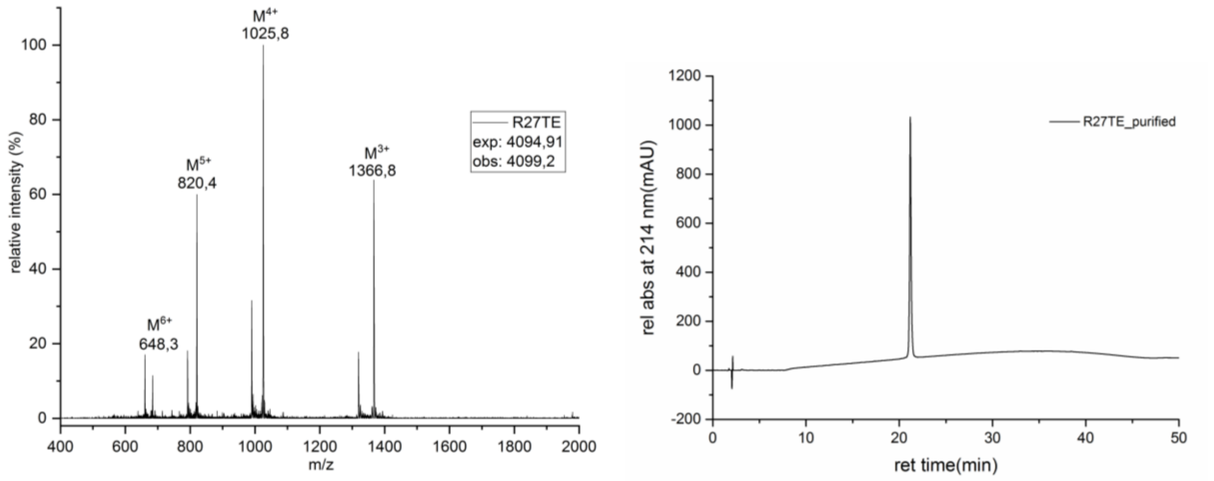

Figure S6. Characterization of the 31-mer R27Apy thioester 3f. a) ESI-MS (Exp: 4096 Da, Obs: 4100 Da) b) analytical RP-HPLC profile

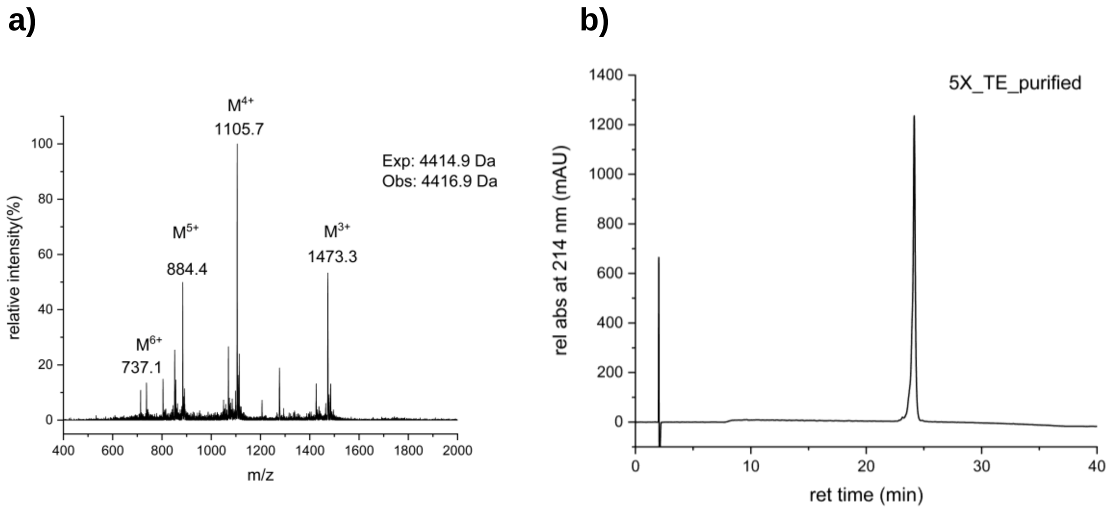

Figure S7. Characterization of the 31-mer 5XApy thioester 3g. a) ESI-MS (Exp: 4415 Da, Obs: 4417 Da) b) analytical RP-HPLC profile

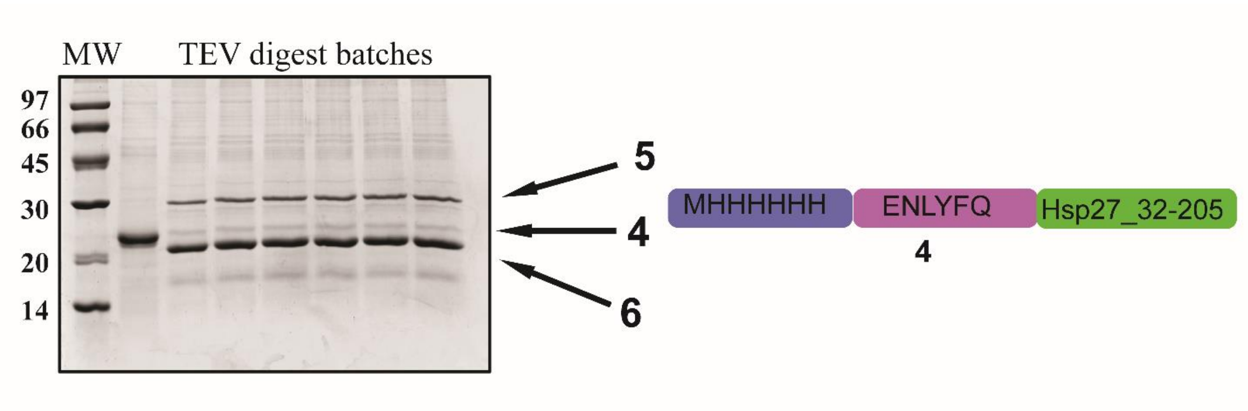

Figure S8. Generation of the recombinantly produced EPL counterpart 6. The generation of **6** via TEV protease **5** mediated scission of a His tag-TEV protease recognition sequence fusion construct **4** was monitered via SDS-PAGE

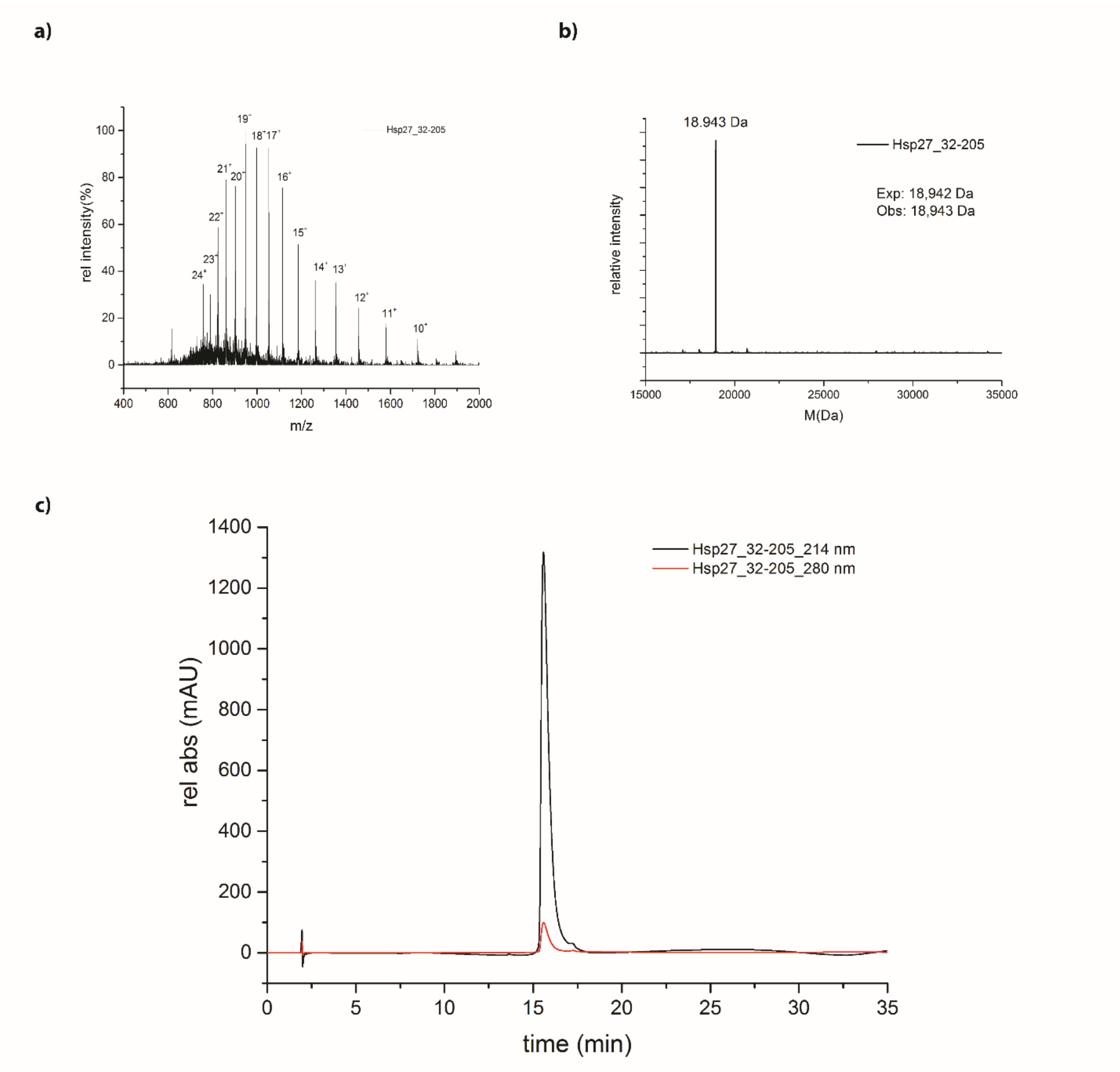

Figure S9. Characterization of the recombinantly produced EPL counterpart 6. a) ESI-MS b) Deconvoluted ESI-MS, exp:18,942 Da, Obs: 18,943 Da c) analytical RP-HPLC profile

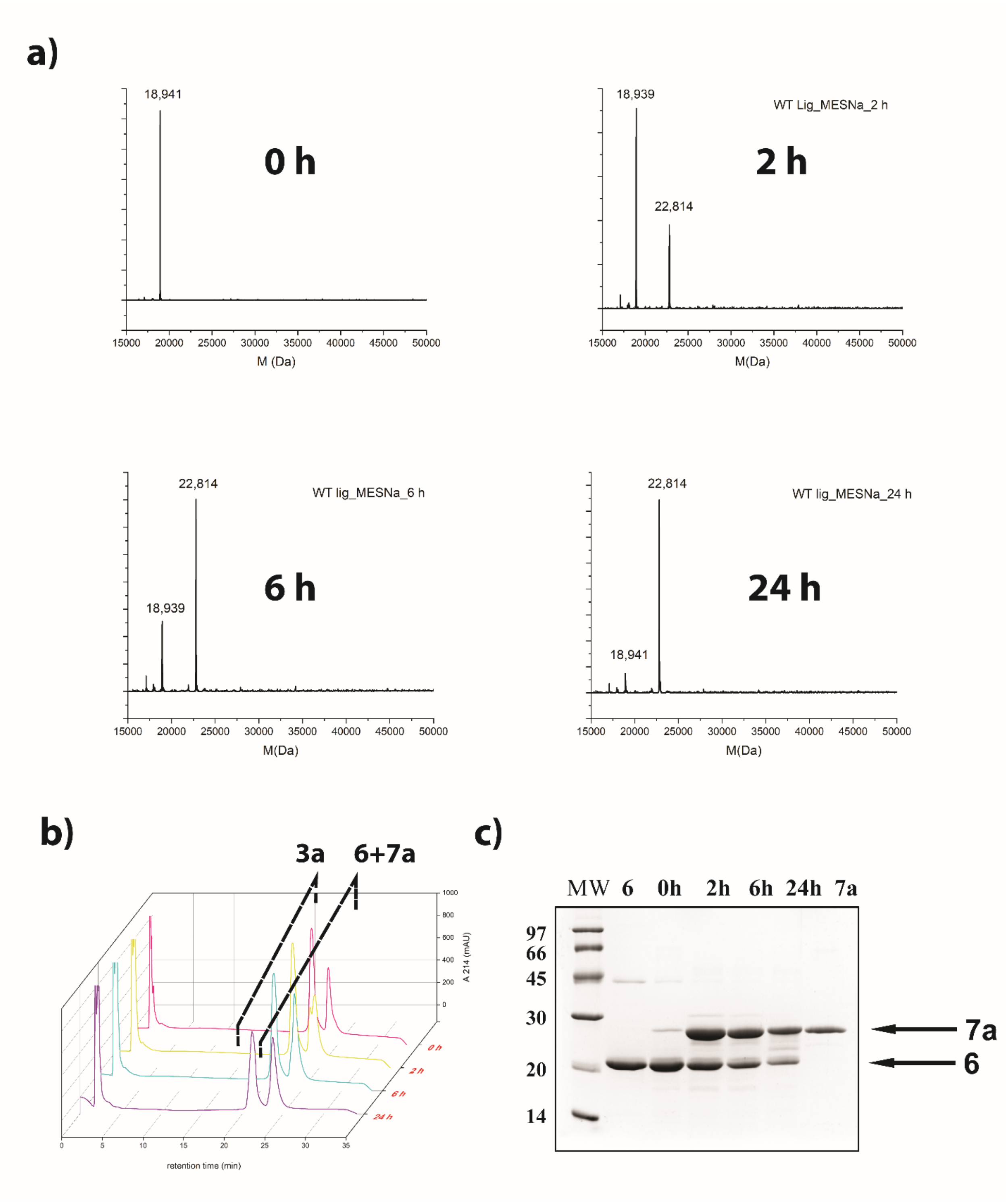

Figure S10. Monitering the MESNa mediated EPL between unmodified thioester 3a and recombinantly produced counterpart 6. a) deconvoluted ESI-MS at indicated time points, exp mass **6**: 18,941 kDa, **7a**: 22,814 kDa b) EPL progress monitored via RP-HPLC c) EPL progress monitored via SDS-PAGE

a)

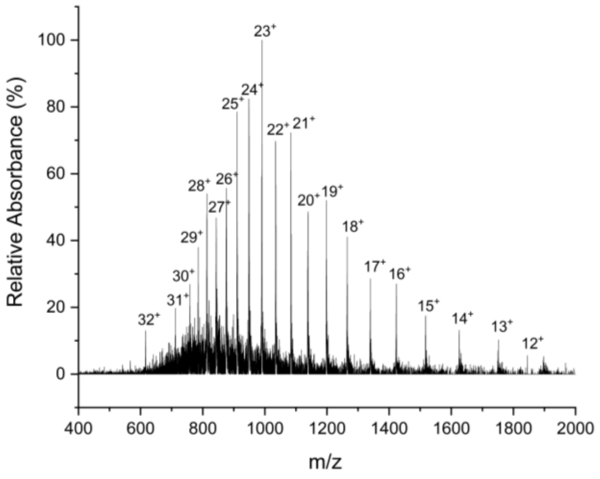

b)

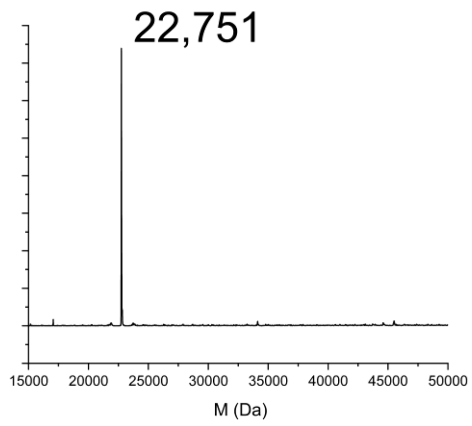

c)

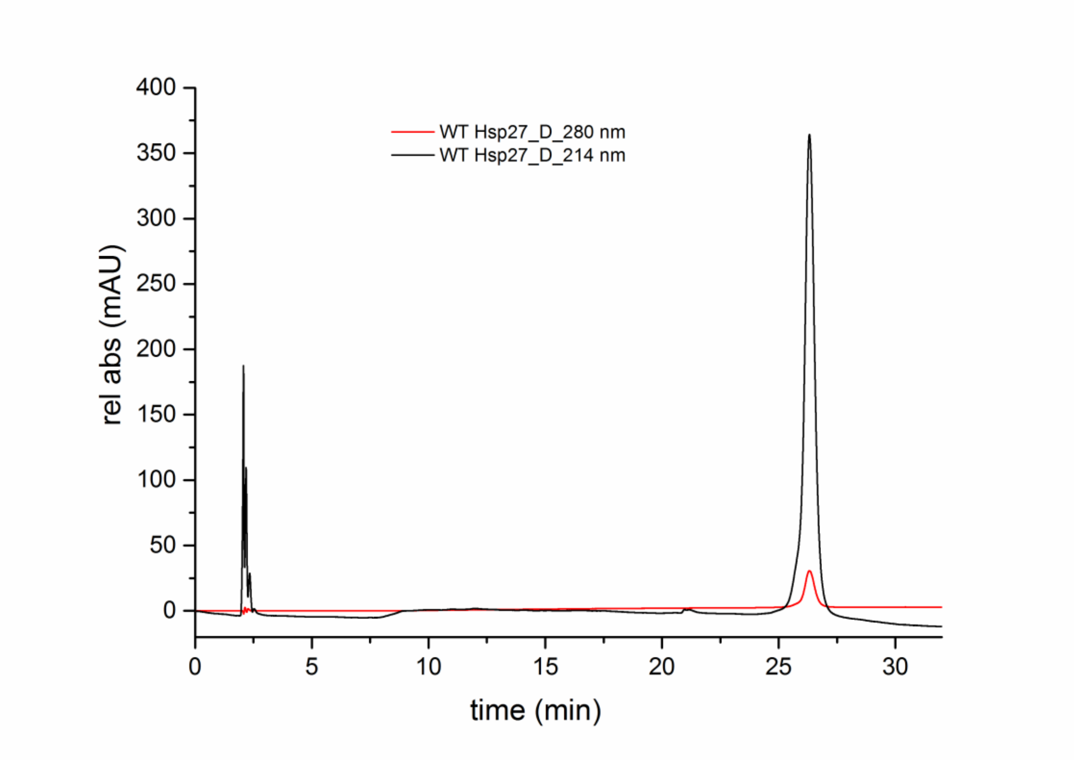

Figure S11. Characterization of the desulfurized and purified ligation product 8a. a) ESI-MS b) Deconvoluted ESI-MS, exp: 22,750 Da, Obs: 22,751 Da c) analytical RP-HPLC profile

a)

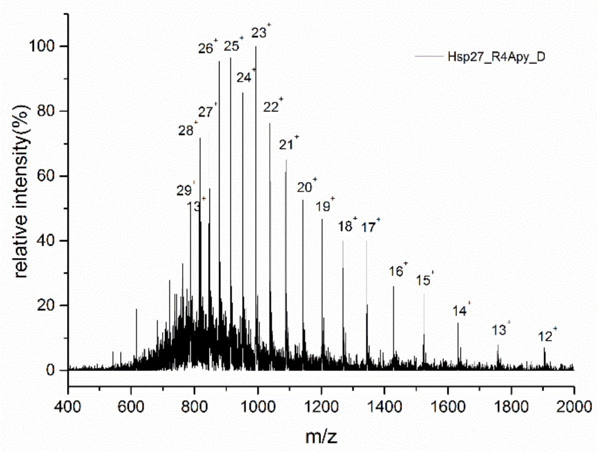

b)

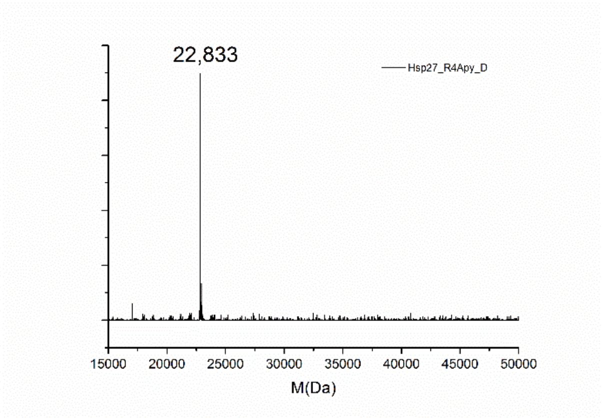

c)

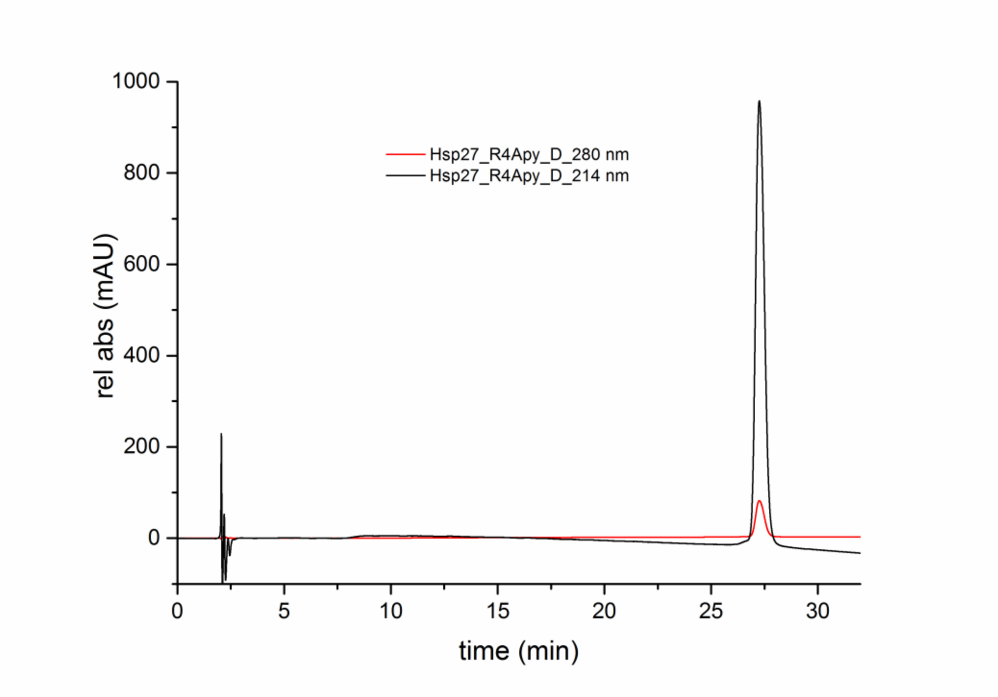

Figure S12. Characterization of the desulfurized and purified ligation product 8b. a) ESI-MS b) Deconvoluted ESI-MS, exp: 22,830 Da, Obs: 22,833 Da c) analytical RP-HPLC profile

a)

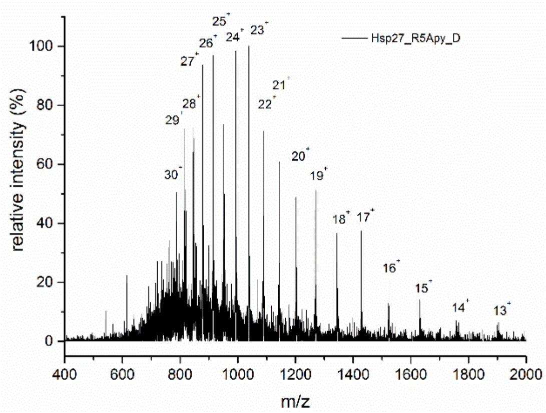

b)

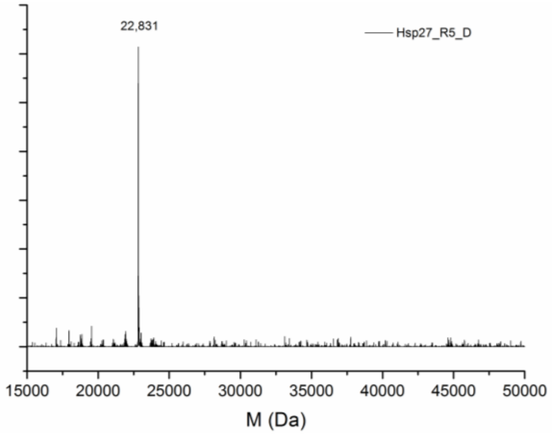

c)

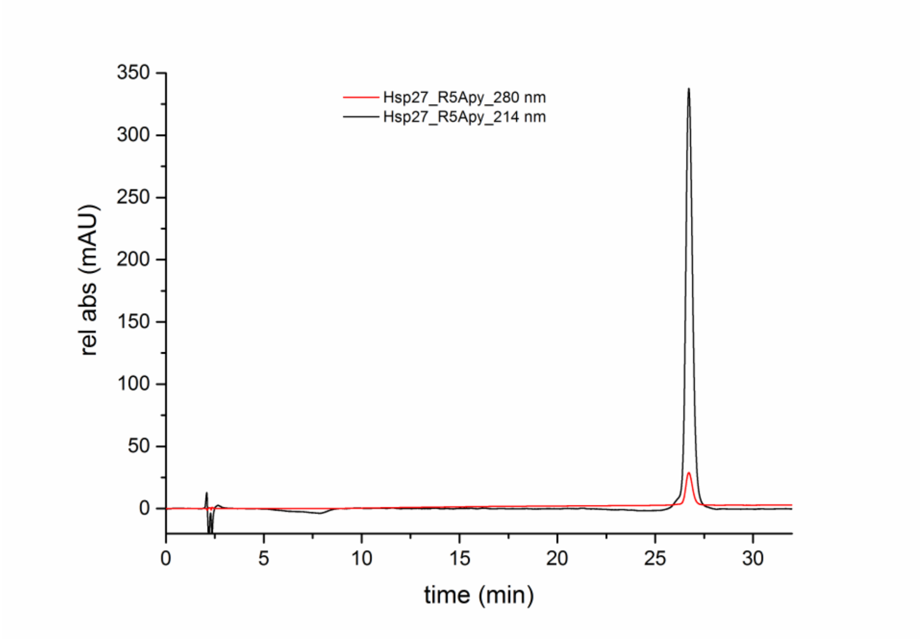

Figure S13. Characterization of the desulfurized and purified ligation product 8c. a) ESI-MS b) Deconvoluted ESI-MS, exp: 22,830 Da, Obs: 22,831 Da c) analytical RP-HPLC profile

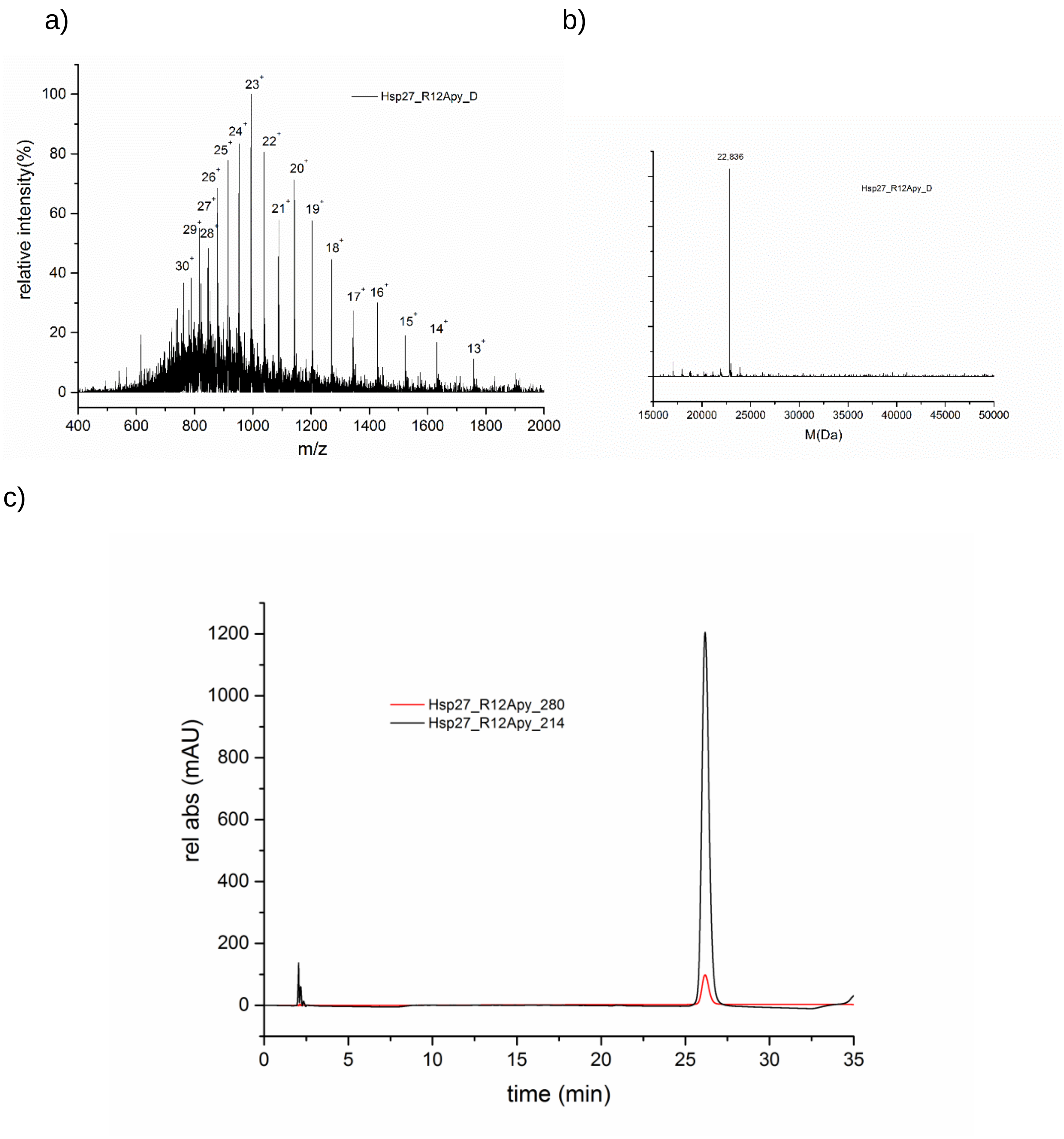

Figure S14. Characterization of the desulfurized and purified ligation product 8d. a) ESI-MS b) Deconvoluted ESI-MS, exp: 22,830 Da, Obs: 22,836 Da c) analytical RP-HPLC profile

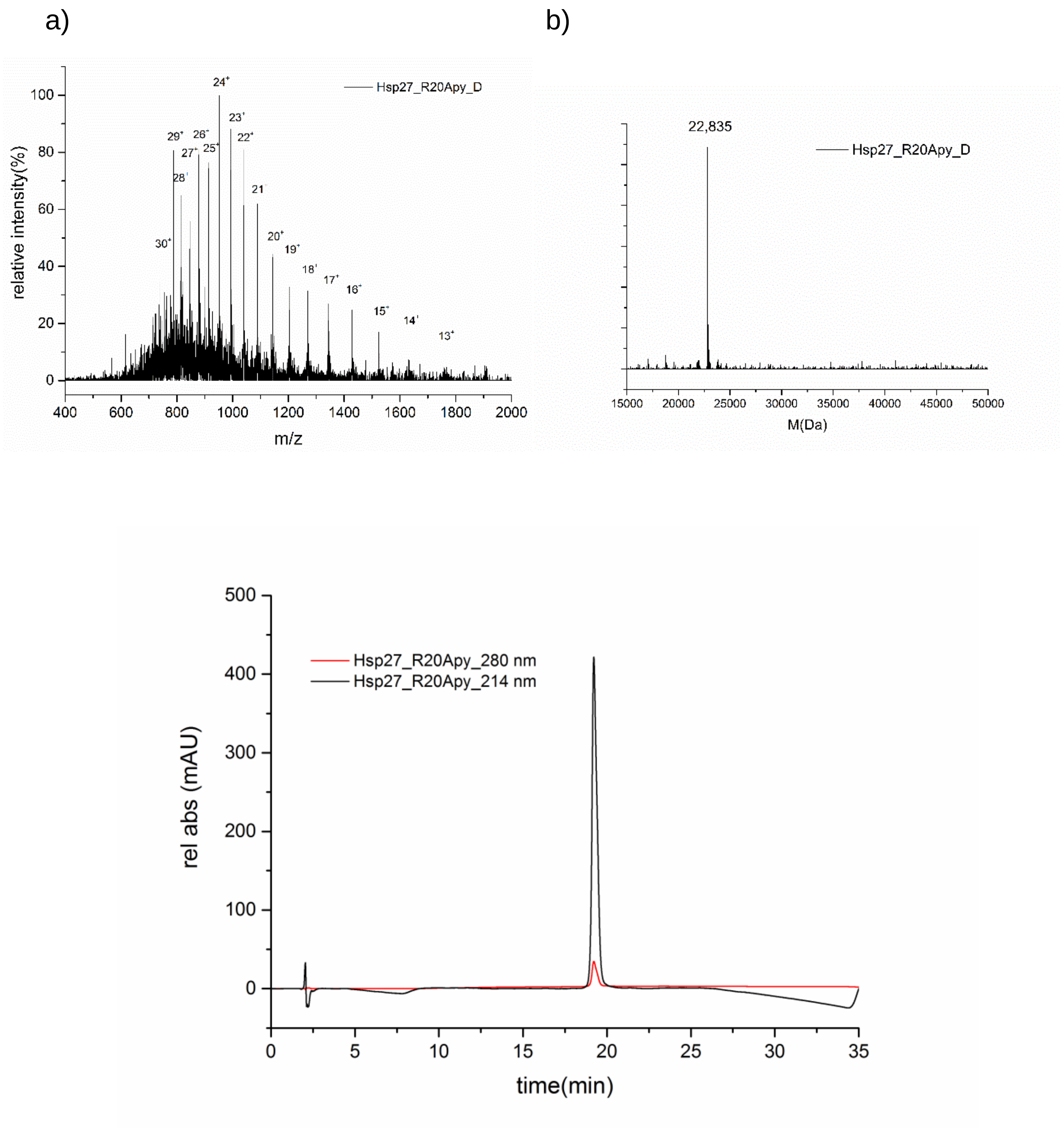

Figure S15. Characterization of the desulfurized and purified ligation product 8e. a) ESI-MS b) Deconvoluted ESI-MS, exp: 22,830 Da, Obs: 22,835 Da c) analytical RP-HPLC profile

a)

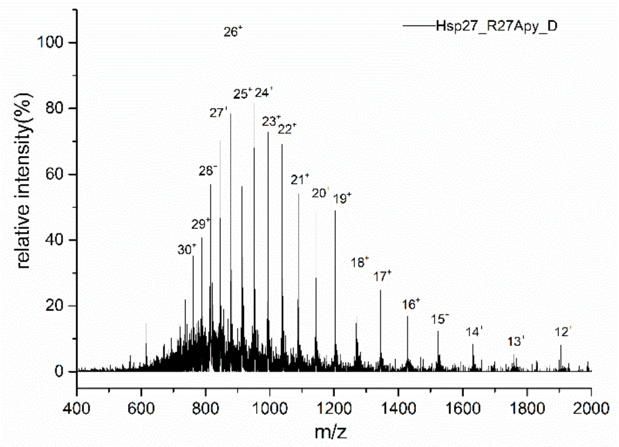

b)

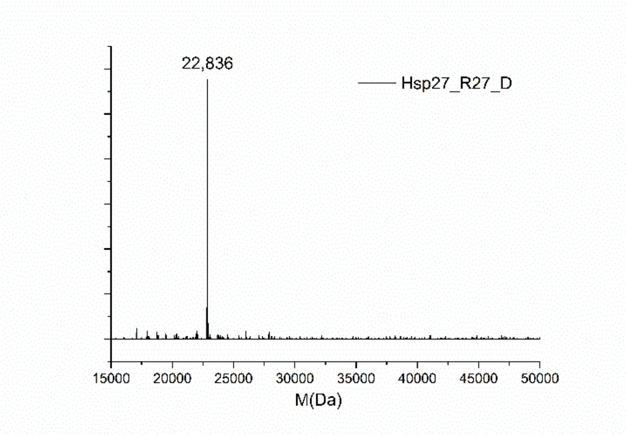

c)

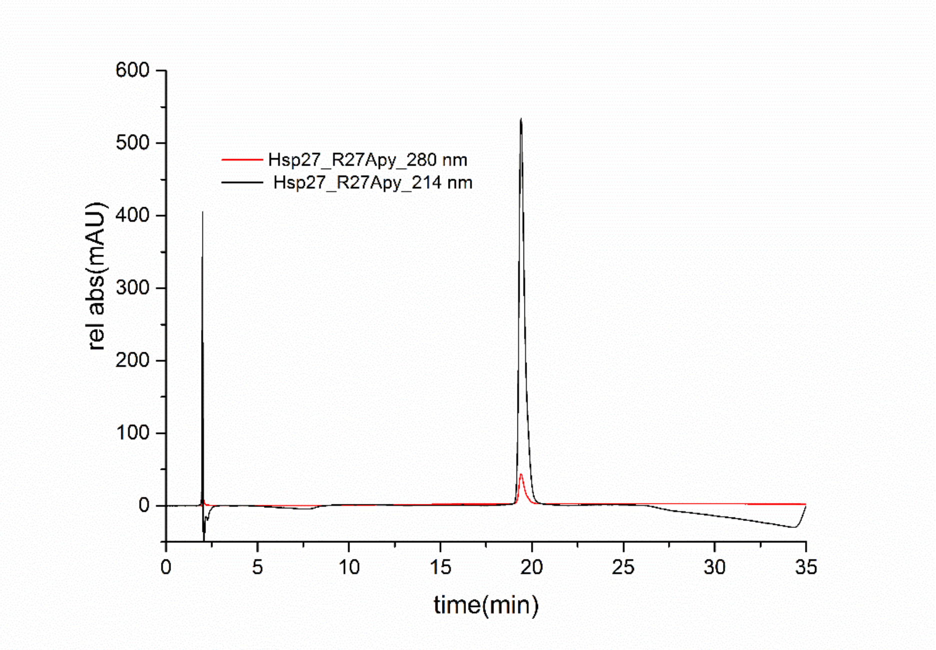

Figure S16. Characterization of the desulfurized and purified ligation product 8f.
a) ESI-MS b) Deconvoluted ESI-MS, exp: 22,830 Da, Obs: 22,836 Da c) analytical RP-HPLC profile

a)

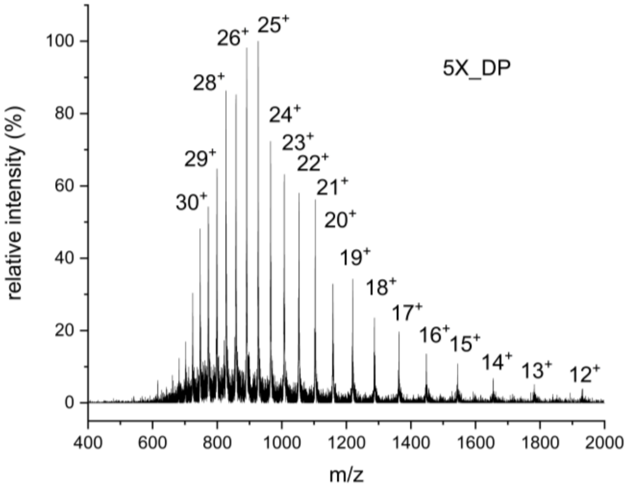

b)

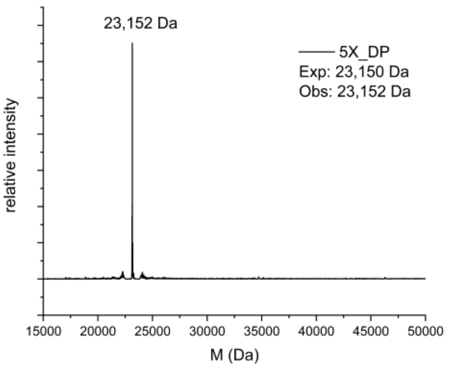

c)

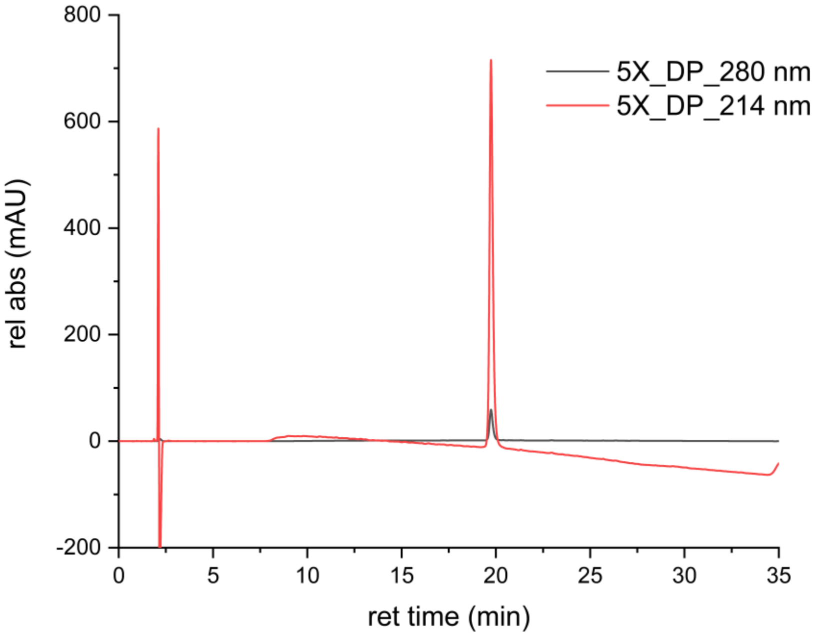

Figure S17. Characterization of the desulfurized and purified ligation product 8g. a) ESI-MS b) Deconvoluted ESI-MS, exp: 23,150 Da, Obs: 23,152 Da c) analytical RP-HPLC profile

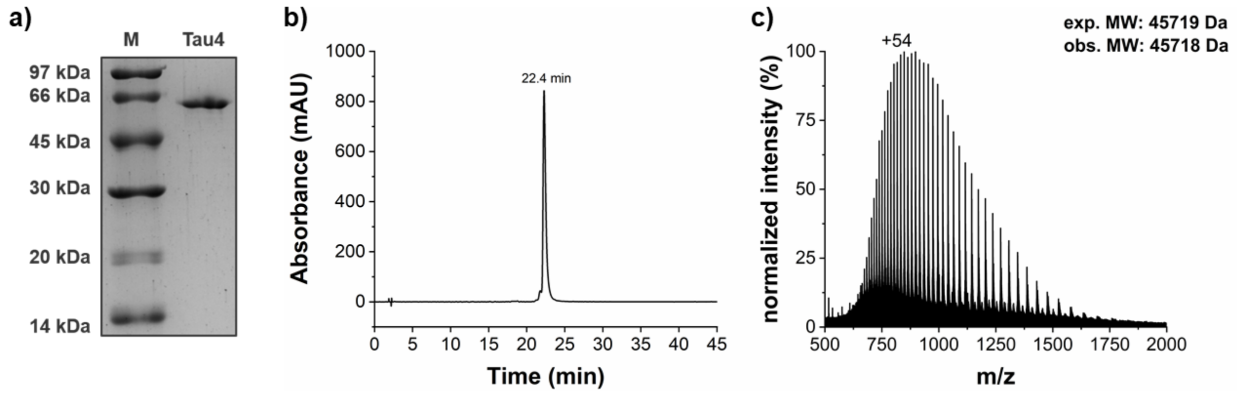

Figure S18. Characterization of the recombinant full-length Tau4. a) SDS-PAGE profile b) analytical RP-HPLC profile c) ESI-MS, exp: 45,719 Da, Obs: 45,718 Da

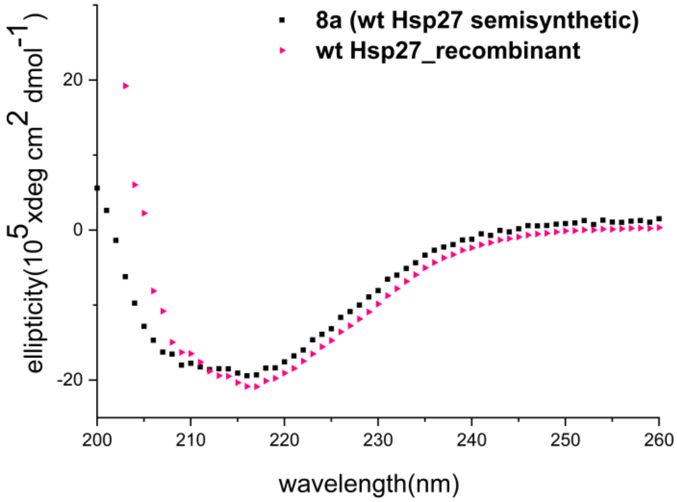

Figure S19. Comparison of the circular dichroism spectra of the semisynthetic wild type Hsp27 (8a) and the recombinantly prepared wild type Hsp27.

Hsp27 WT sequence:

MTERRVPFSLLRGPSWDPFRDWYPHSRLFDQAFGLPRLPEEWSQWLGGSS
WPGYVRPLPPAAIESPAVAAPAYSRALSRQLSSGVSEIRHTADRWRVSLD
VNHFAPDELTVKTKDGVVEITGKHEERQDEHGYISRCFTRKYTLPPGVDPTQVSSSLSPE
GTLTVEAPMP KLATQSNEIT IPVTFESRAQ LGGPEAAKSDETAAK

Synthetic gene sequence for the expression of fusion construct 4 (codon optimized)

CAT ATG CAT CAT CAT CAC CAC CAT GAG AAC CTG TAC TTC CAG TGC TTT GGC CTG CCT CGT CTG CCC GAA GAA TGG TCG CAG TGG TTG GGA GGG AGT TCA TGG CCA GGC TAT GTG CGT CCG TTA CCG CCT GCT GCG ATT GAG TCT CCA GCC GTT GCA GCT CCG GCC TAT AGC CGC GCA CTT AGT CGT CAG CTG AGC TCT GGC GTA TCC GAG ATT CGC CAT ACT GCG GAT CGT TGG CGC GTT AGC CTG GAT GTC AAC CAC TTT GCG CCC GAT GAA CTG ACG GTC AAA ACC AAG GAT GGT GTG GTG GAA ATC ACG GGC AAA CAC GAA GAG CGG CAA GAC GAA CAT GGG TAC ATC TCG CGC TGT TTT ACC CGC AAG TAT ACC CTC CCT CCG GGT GTA GAC CCG ACA CAG GTT TCC AGC TCC TTA TCA CCG GAA GGC ACC CTG ACC GTC GAA GCT CCA ATG CCG AAA CTG GCC ACT CAG TCG AAT GAG ATC ACC ATT CCG GTG ACG TTC GAA AGT CGC GCA CAA TTG GGT GGT CCG GAA GCG GCG AAA AGC GAC GAA ACA GCC GCG AAA TAA CTC GAG

Translated (amino acid sequence of the fusion construct 4 used in this study)

MHHHHHHENLYFQCFGLPRLPEEWSQWLGGSSWPGYVRPLPPAAIESPAVAAPAYSRA LSRQLSSGVSEIRHTADRWRVSLDVNHFAPDELTVKTKDGVVEITGKHEERQDEHGYIS RCFTRKYTLPPGVDPTQVSSSLSPEGTLTVEAPMPKLATQSNEITIPVTFESRAQLGGPEA AKSDETAAK

Rererences

1. Matveenko, M., and Becker, C. F. W. (2017) Synthetic Approach to Argpyrimidine as a Tool for Investigating Nonenzymatic Posttranslational Modification of Proteins, *Synlett 28*, 1950-1955.
2. Ellmer, D., Brehs, M., Haj-Yahya, M., Lashuel, H. A., and Becker, C. F. W. (2019) Single Posttranslational Modifications in the Central Repeat Domains of Tau4 Impact its Aggregation and Tubulin Binding, *Angew. Chem. Int. Ed. 58*, 1616-1620.
3. Mymrikov, E. V., Daake, M., Richter, B., Haslbeck, M., and Buchner, J. (2017) The Chaperone Activity and Substrate Spectrum of Human Small Heat Shock Proteins, *J. Biol. Chem. 292*, 672-684.
4. Lefèvre, J., Chernov, K. G., Joshi, V., Delga, S., Toma, F., Pastré, D., Curmi, P. A., and Savarin, P. (2011) The C Terminus of Tubulin, a Versatile Partner for Cationic Molecules, *J. Biol. Chem. 286*, 3065-3078.
